# Transcriptomic divergence between upland and lowland ecotypes contributes to rice adaptation to a drought‐prone agroecosystem

**DOI:** 10.1111/eva.13054

**Published:** 2020-07-16

**Authors:** Zhi Luo, Jie Xiong, Hui Xia, Xiaosong Ma, Min Gao, Lei Wang, Guolan Liu, Xinqiao Yu, Lijun Luo

**Affiliations:** ^1^ College of Plant Sciences & Technology Huazhong Agricultural University Wuhan China; ^2^ Shanghai Agrobiological Gene Center Shanghai China

**Keywords:** breeding, drought tolerance, expression divergence, *Oryza sativa*, transcriptomic adaptation, upland rice

## Abstract

**Introduction:**

Transcriptomic divergence drives plant ecological adaptation. Upland rice is differentiated in drought tolerance from lowland rice during its adaptation to the drought‐prone environment. They provide a good system to learn the evolution of drought tolerance in rice.

**Methods and Results:**

We estimate morphological differences between the two rice ecotypes under well‐watered and drought conditions, as well as their genetic and transcriptomic divergences by the high‐throughput sequencing. Upland rice possesses higher expression diversity than lowland rice does. Thousands of genes exhibit expression divergences between the two rice ecotypes, which contributes to their morphological differences in drought tolerance. These transcriptomic divergences contribute to drought adaptation of upland rice during its domestication. Mutations in transcriptional regulatory regions, which cause presence and absence of *cis*‐elements, are the cause of expression divergence. About 15.3% transcriptionally selected genes also receive sequence‐based selection in upland or lowland ecotype. Some highly differentiated genes promote the transcriptomic divergence between rice ecotypes *via* gene co‐expression network. In addition, we also detected transcriptomic trade‐offs between drought tolerance and productivity.

**Discussion:**

Many key genes, which promote transcriptomic adaptation to drought in upland rice, have great prospective in breeding water‐saving and drought‐resistant rice. Meanwhile, appropriate strategies are required in breeding to overcome the potential transcriptomic trade‐off.

## 1 INTRODUCTION

Rice is one of the most important cereal foods and feeds more than 50% of global populations. Elite paddy rice requires great amount of water and is sensitive to drought (Luo, 2010). Drought is therefore the most severe limiting factor in rice production (Bernier, Atlin, Serraj, Kumar, & Spaner, 2008). Given the increasing water shortage and frequent droughts, improvement for drought tolerance in rice has become a primary breeding target (Bernier et al., 2008; Luo, 2010; Luo et al., 2019). However, drought tolerance in rice is a complex quantitative trait underlain by several mechanisms (Bernier et al., 2008; Luo, 2010), cross‐talk among plant hormones (Verma, Ravindran, & Kumar, 2016), and complex gene networks (Hadiarto & Tran, 2011). The improvement of drought tolerance by a single gene still has limited success in fields, although many drought‐tolerant genes have been functionally characterized in laboratories (Fang & Xiong, 2015). Therefore, systematic solutions are required to improve drought tolerance in rice (Khan, Sovero, & Gemenet, 2016; Todaka, Shinozaki, & Yamaguchi‐Shinozaki, 2015), which makes it essential to understand the evolutionary process of drought tolerance during domestication.

Upland and lowland rice are the two most important ecotypes that are domesticated in agroecosystems under contrasting soil–water conditions (Bernier et al., 2008). They have been reported to be morphologically and genetically differentiated in terms of drought resistance (Bernier et al., 2008; Lyu et al., 2014; Xia et al., 2019), which makes them an ideal system for investigating the evolutionary process of drought resistance in rice. There have been several studies on genetic divergence between upland and lowland rice related to drought resistance (Lyu et al., 2014; Xia et al., 2014, 2019). They suggest the ecological adaptation between upland and lowland rice refers to limited genes and/or regions across the rice genome (Lyu et al., 2014; Xia et al., 2019). Meanwhile, it has been reported that there are thousands of drought‐responsive genes in rice (Ma et al., 2016; Zhang, Li, & Xiao, 2016). These drought‐responsive genes shall contribute a lot to rice drought adaptation and drought tolerance, which may result in the expression divergence between the two rice ecotypes.

Expression divergence, resulted from positive selection on the gene expression (Shi et al., 2012; Skinner, Mohan, Haque, Zhang, & Savenkova, 2012), has great significance in speciation (Guo et al., 2016; Pavey, Collin, Nosil, & Rogers, 2010), adaptation (Dayan, Crawford, & Oleksiak, 2015; Lasky et al., 2014), and domestication (Koenig et al., 2013; Liu et al., 2015). Many studies have indicated that the adaptive evolution of a complex trait is a systematic process at the transcriptome scale that involves large number of genes (Dayan et al., 2015; Gould, Chen, & Lowry, 2018; Liu et al., 2015). Given many successes in transcriptomic explanations of complex adaptive traits (Dayan et al., 2015; Gould et al., 2018), the investigation of transcriptomic divergence among the two rice ecotypes differing in drought tolerance can be an effective method to explore the molecular bases of drought tolerance and its evolution in rice.

In this study, we investigate the transcriptomic divergence between genetic differentiated upland and lowland rice ecotypes, as well as their genetic divergence. We try to address whether there has been a transcriptomic divergence between the upland and lowland rice ecotypes, and whether the transcriptomic divergence contributes to better drought adaptation in upland rice?

## 2 MATERIALS AND METHODS

### 2.1 Plant materials

We chose 124 rice genotypes, which contains 40 typical *Geng* (*japonica*) upland and 50 typical *Geng* (*japonica*) lowland landraces, from China to investigate their morphological and transcriptomic divergence in well‐watered and drought‐treated (DT) fields (Table S1). These upland and lowland landraces are genetically differentiated and ideal for study differentiation between the two rice ecotypes (Xia et al., 2019). The typical upland and lowland landraces were determined by the results of populations structure inference and principal component analysis described below, in which the typical upland and lowland landraces were clearly seperated.

### 2.2 Evaluation of agronomic and drought tolerance traits in landraces and modern varieties under drip‐watered (CK) and DT conditions

A field evaluation of agronomic and drought tolerance traits of landraces was conducted in the drought resistance screening facility at Baihe Experimental Station, Shanghai, in 2016 (May to October). The canopy of the facility can be closed on rainy days to enable continuous drought conditions. The depth of the soil layer in the experimental field was limited to 30 cm by a nylon membrane, which restricted root development. Therefore, the aboveground drought tolerance by osmotic adjustment and antioxidant capacity could be well estimated, while differences in drought avoidance by roots could be largely mitigated. Plants of each rice genotype were planted in two nearby fields: One was treated with drought (DT) during mid‐ to later periods (from the tillering stage to the heading stage), and the other remained well‐watered by drip irrigation during drought and served as the control (CK). Due to the limitation of area of the drought resistance screening facility, rice seedlings of each genotype were transplanted into a plot with 8 rows × 8 hills at 18‐cm intervals on the 15th of June, 30 days after germination. Both the DT and CK fields were normally irrigated as paddy fields during the first 20 days after transplanting. After the seedlings began tillering (5th of July), water was pumped from the DT field to let it dry naturally. Drip irrigation was applied to the CK field to maintain its normal soil–water content. After 40 days of DT, when the soil–water content at a 30‐cm depth dropped to ~12.6% and all rice genotypes showed signs of leaf‐rolling, the drought treatment was stopped, and both fields were re‐irrigated. Ten agronomic traits, namely, plant height (PH), number of tillers (NT), flag leaf length (FLL), flag leaf width (FLW), number of grains (NG), 100‐grain weight (100GW), grain yield per plant (GY), aboveground biomass, the harvest index (HI), and fecundity, were measured in the DT and CK fields when rice plants were matured or after harvest. These agronomic traits were measured on six individuals in the middle of the plot to avoid the border effect. Relative water content (RWC) under drought and relative grain yield (RGY) (grain yield in DT/grain yield in CK) were measured to estimate drought resistance. RWC was measured from three replicates of top leaves on the 5th of August when genotypes in the DT field represented differences in leaf‐rolling. The RWC was calculated as follows: (weight of fresh leaf‐weight of dry leaf)/(weight of saturated leaf‐weight of dry leaf). To ensure the result of morphological divergence between the two ecotypes is repeatable, we replicated the field experiment in 2017 (May–October) at Baihe Experimental Station, Shanghai. As expected, the general result from comparisons between upland and lowland rice ecotypes in 2017 was similar with that in 2016 (Xia et al., 2019).

### 2.3 RNA sequencing (RNA‐seq) to quantify gene expression

Leaf samples were collected on the same day that the RWC was measured. Total RNA of each rice genotype was extracted from mixed leaf tissue from three individuals using TRIzol® reagent following the manufacturer's instructions (Invitrogen), and genomic DNA was removed using DNase I (TaKaRa). RNA‐seq libraries were constructed following the TruSeq™ RNA sample preparation kit (Illumina, San Diego, CA). Paired‐end libraries were sequenced by an Illumina X Ten platform (2 × 151‐bp read length). The raw paired‐end reads were quality controlled by Trimmomatic with default parameters (http://www.usadellab.org/cms/uploads/supplementary/Trimmomatic). Then, clean reads were separately aligned to reference genome (https://rapdb.dna.affrc.go.jp/download/irgsp1.html) with orientation mode using hisat2 (https://ccb.jhu.edu/software/hisat2/index.shtml) software. This software was used to map with default parameters. The basic information of RNA‐seq for all samples was provided in Table S2. We then used htseq to count each gene reads (https://htseq.readthedocs.io/en/release_0.11.1/). R statistical package edgeR (Empirical analysis of Digital Gene Expression in R, http://www.bioconductor.org/packages/release/bioc/html/edgeR.html/) was used to identify differentially expressed genes (DEGs) between the CK and DT conditions (also coded as drought‐responsive genes, DRGs) using the following criteria: (a) a log_2_ (fold change) >1 or <−1 and (b) a *p* value < 0.05. Six drought‐resistant genes were selected, and their expression in ~30 samples was validated, for which *Actin* was used as the reference (Table S3). Gene expression values calculated with RNA‐seq were well validated by the expression values obtained with quantitative PCR (qPCR), as shown by their significant correlations (Figure S1).

### 2.4 Resequencing to call single nucleotide polymorphisms (SNPs)

Genomic DNA of each genotype was extracted from rice seedlings using an E.Z.N.A. Tissue DNA kit (Omega Bio‐Tek). Paired‐end libraries (150 bp*2) were prepared following Illumina's standard genomic DNA library preparation procedure. Samples were sent for resequencing by an Illumina X Ten platform. Sickle (https://github.com/najoshi/sickle) was applied to perform read trimming with the default parameters to obtain clean data. The high‐quality reads were then aligned to the IRGSP‐1.0 reference genome (https://rapdb.dna.affrc.go.jp/download/irgsp1.html) using BWA (http://bio‐bwa.sourceforge.net/) software with “bwa aln” mode (Li & Durbin, 2009). The valid BAM file was used to detect SNPs and short insertions/deletions (indels) by the GATK “UnifiedGenotyper” (http://www.broadinstitute.org/gatk/) (McKenna et al., 2010). Variant call format (VCF) files were generated by quality filtering (VariantFiltration with the following parameters: QD < 2.0||FS > 60.0||MQ < 40.0||SOR > 10.0). Furthermore, VCF files were filtered with VCFtools (version 0.1.11, parameters: ‐‐minQ 20 ‐‐minDP 4) (Danecek et al., 2011). The annotation of detected variations was performed by ANNOVAR (http://www.openbioinformatics.org/annovar/) (Wang, Li, & Hakonarson, 2010). We selected three cis‐element‐altering SNPs in the gene *Os06g0269200* to validate SNPs generated from resequencing *via* the Sanger method (Table S3, Figure S2). This result indicated the *cis*‐element‐altering SNPs called from resequencing were reliable.

### 2.5 Data analysis

#### 2.5.1 Comparison of agronomic and drought‐tolerant traits between upland and lowland ecotypes

Any significant differences in the measured traits between ecotypes were detected by an independent *t* test. We also compared these traits between the DT and CK conditions by a paired *t* test. The independent and paired *t* tests were applied between (a) total upland and lowland genotypes, and (b) typical upland and lowland genotypes. The difference in each trait between total upland and lowland ecotypes was quantified by the quantitative genetic divergence (*Q*_ST_). The *Q*_ST_ of each trait was calculated as *Q*_ST_ = *V*_B_/(*V*_B_ + 2V_W_), where *V*_B_ was the variance between ecotypes and *V*_w_ was the variance within ecotypes. The variation of *Q*_ST_ was estimated by 1,000 permutations. We then compared the *Q*_ST_ and neutral genomic *F*_ST_ calculated from intergenic SNPs.

#### 2.5.2 Population structure inference

Three analyses were applied to infer the population structure among 124 rice genotypes. First, principal component analysis (PCA) was conducted using genomic SNPs (minor allele frequency >5%, 2,382,247 SNP) called from the resequencing data. Second, genetic distances among the 124 genotypes were calculated, and EMBOSS/fneighbor was used to build a neighbor‐joining (NJ) phylogenetic tree (http://emboss.toulouse.inra.fr/cgi‐bin/emboss/fneighbor?_pref_hide_optional=1). Finally, the web program fastStructure (https://github.com/rajanil/fastStructure) was used to perform population structure analysis, applying the maximum likelihood method. Based on the results of PCA, NJ cluster, and population structure, we can determine typical upland and lowland genotypes in which they were clearly separated.

#### 2.5.3 Calculation of genetic diversity (π) and genetic divergence (*F*_ST_) for each gene

Genetic diversity (π) was calculated for each gene (upstream 2,000 bp + gene body + downstream 2,000 bp) in upland, lowland, and total landraces based on the resequencing data. The genetic divergence (*F*_ST_) between the two types of ecotypes was also calculated for each gene. We further used the single‐sample Kolmogorov–Smirnov test to determine whether the distribution of highly differentiated genes (gene density) obeys the uniform distribution among 5000 kb windows across the rice genome via SPSS ver.19.0.

#### 2.5.4 Estimation of expression variation and expression divergence

To estimate gene expression variation, the expression diversity (*E_d_*) for each gene was calculated among the total genotypes, upland landraces, and lowland landraces. *E_d_* was calculated as $E_{d}=\frac{\sum_{i=1}^{n}\left|E_{i}‐E_{p}\right|}{\left(n‐1\right)E_{p}}$, where *n* represents the number of individuals, *E_i_* represents the FPKM of a given gene of the *i*th individual in the population, and *E_p_* represents the mean expression of a given gene (Xu et al., 2016). We also calculated the *Q*_ST_ for the expression of each gene to quantify its expression divergence. We can then perform correlation analysis between the *F*_ST_ and *Q*_ST_ among all genes to test whether the genetic divergence has any impact on expression divergence.

#### 2.5.5 Determination of expressed genes under directional selection

Genes with low intraspecies variation but high interspecies divergence are under directional selection (Romero, Ruvinsky, & Gilad, 2012). To make the outcome of expression divergence generated between the two ecotypes more reliable, 40 and 50 typical upland and lowland rice genotypes (Table S1) were chosen in this analysis. We applied a rank‐based method to identify the transcriptionally selected genes (TDSGs), which receives the directional selection at the transcriptional level, following the rationale of Guo et al. (2016). Briefly, we applied an independent samples *t* test to identify DEGs between rice ecotypes (*p* < .05), which were considered genes with high interspecies divergence. We determined the threshold for genes with low between‐individual variance within ecotypes using the method by Blekhman, Oshlack, Chabot, Smyth, and Gilad (2008). Briefly, the between‐individual variance (*y*) of each gene was estimated by a linear mixed model (*y* = *ax*_1_ + *bx*_2_ + c) accounting for origin (*x*_1_) and ecotype effects (*x*_2_) as random factors and in which c was the random error. We first generated a distribution of ranked between‐individual variances. According to the distribution, we fitted two straight lines to two linear parts of the distribution, for which the first linear part was modeled using the top 1% of genes and the latter linear part was modeled using the bottom 80% of genes. Then, we set the intersection point of the lines as the cutoff for low between‐individual variance. We also calculated the selection index (SI) (Warnefors & Eyre‐Walker, 2012) for all detected genes under transcriptionally directional selection. The SI is an extension of the widely used McDonald–Kreitman test, in which the within‐species variances (*V*_w_) and between‐species variances (*V*_b_) of gene expression are compared with the within‐species polymorphism (*π*) and between‐species divergence (*D*_xy_) of neutrally evolving sequences (intron sequences from the resequencing). A positive SI value for a gene indicates that its expression is under directional selection (Warnefors & Eyre‐Walker, 2012).

#### 2.5.6 Testing the association of transcriptomic divergences with morphological divergences and rice drought adaptation

Upland and lowland rice ecotypes adapt to environments with different factors (soil–water conditions, soil–nutrient conditions, temperatures, etc.). Fisher's exact test was applied to test whether genes responding to a given environmental factor were enriched by TDSGs. Drought‐responsive genes (DRGs) were defined as those (a) detected as DEGs (Log_2_^(fold change)^>1 or <−1 and *p* < .05) between the CK and DT conditions in at least one rice genotype and (b) having significant differences (*p* < .05) in expression between samples from the CK and DT fields based on a paired *t* test. Genes responding to other environmental factors were extracted from previous transcriptomic studies and listed in Table S4.

Correlation analyses (Pearson's correlation coefficient) were conducted between gene expression (quantified by FPKM) and measured traits among typical genotypes under the CK and DT conditions, respectively. A gene whose expression was significantly correlated (*p* < .05) with an agronomic trait (e.g., NT) was defined as a trait‐correlated (e.g., NT‐correlated) gene. We calculated the ratio of trait‐correlated genes in TDSGs as follows: (no. of genes correlated with a trait in TDSGs/total no. of TDSGs). We applied the correlation analysis between the ratio of genes correlated with a trait in TDSGs and its *Q*_ST_ value of measured traits. Significant correlations between these values suggested that transcriptomic divergences contributed to morphological divergences between ecotypes.

RWC is a featured trait of drought tolerance, while NT is an important agronomic trait. These traits were morphologically differentiated based on *F*_ST_‐*Q*_ST_ comparisons. Upland rice possesses less tillers in CK but higher RWC in DT, while lowland rice possesses more tillers but lower RWC in DT. It means a balance exists between NT and drought tolerance during rice domestication. We thus selected the two traits for testing potential transcriptomic trade‐offs between drought tolerance and tillering ability. If the expression of a gene was oppositely correlated with RWC‐DT and NT‐CK (e.g., positively correlated with RWC‐DT but negatively correlated with NT‐CK, or vice versa), this gene was assumed to have a trade‐off between rice drought tolerance and tillering ability. For more cautious, these genes with FDR < 0.05 were defined as RWC‐DT and NT‐CK correlated genes. We further performed Fisher's exact test to examine whether genes with or without NT‐RWC trade‐offs tended to be transcriptionally selected.

If the transcriptomic divergence contributes to rice drought tolerance, the expression of a D‐TDSG in upland rice should help it to maintain better water status, which was reflected by RWC‐DT. Based on our data, two types of RWC‐correlated genes could be beneficial for the adaptation of upland rice to drought: (a) a positively RWC‐correlated gene possessing a higher expression level in upland rice, and (b) a negatively RWC‐correlated gene exhibits a lower expression level in upland rice. We further applied Fisher's exact test to determine whether genes of these two kinds of RWC‐correlated genes were significantly enriched by in D‐TDSGs.

#### 2.5.7 Identification of genes under directional selection based on resequencing data

To explore potential associations of transcriptomic divergence with genetic divergence, we conducted correlation analyses between expression divergence (*Q*_ST_) and genetic divergence (*F*_ST_) as well as between *E_d_* and π. Moreover, the signature of recent directional selection was detected based on the distribution of the ratios of polymorphism within an ecotype (π) to between‐ecotype divergence (*D*_xy_). Genes with a small π but large *D*_xy_ are likely under directional selection. We considered the genes in the lowest 5% tail of the polymorphism/divergence ratio (π/*D*_xy_) distribution as having been under recent directional selection (Holloway, Lawniczak, Mezey, Begun, & Jones, 2007). Genes under sequence‐based directional selection in upland and lowland ecotypes were coded as U‐DSG and L‐DSG, respectively.

Variation in transcription regulatory regions, particularly in *cis*‐regulatory elements, contributes greatly to gene expression variation and divergence (Holloway et al., 2007; Lovell et al., 2016). We defined *cis*‐element‐altering SNPs, which could cause the formation or loss of *cis*‐elements in the regulatory region (from the upstream 2,000 bp to the transcription start site) of a gene. One hundred and two cis‐elements from the PLACE database, which play a role in plant stress and hormone responses, were involved in this study (Table S5). To test whether the mutations in *cis*‐elements have impacts on gene expression, the *Q*_ST_ of a gene between ecotypes or between haplotypes was calculated with 1,000 permutations. If the *Q*_ST_ value between haplotypes was significantly greater than that between ecotypes, potential influences of *cis*‐element‐altering SNPs on gene expression were confirmed. Eleven hub highly differentiated genes (HDGs) identified by weighted gene co‐expression network analysis (WGCNA) were included in this analysis. The haplotype of each gene was determined by its *cis*‐element‐altering SNPs (Table S6).

#### 2.5.8 Gene ontology (GO) analyses of five different types of genes

We conducted analyses of GO enrichment for the five types of genes: HDG (beyond the 95% confidence interval of *F*_ST_ among total genes), CK‐/ D‐TDSG (transcriptionally selected genes in CK and DT using the rank‐based method modified by Guo et al., 2012), and U‐/ L‐DSG (genes under sequence‐based directional selection in upland and lowland rice using the method proposed by Holloway et al., 2007) using the software GOATOOLS (https://github.com/tanghaibao/GOatools) (Klopfenstein et al., 2018). The top 20 GO terms of biological processes (based on p value) are listed and compared among different types of genes.

#### 2.5.9 Gene expression network analysis (WGCNA)

It has been suggested that hypothesize that key genes in the co‐expression network are the potential driving forces of plant transcriptome adaptation (Filteau, Pavey, St‐Cyr, & Bernatchez, 2013; Pavey et al., 2010). Learning the role of gene networks in rice transcriptomic adaptation to drought can deepen our systematic understanding of the formation of drought tolerance in upland rice. For this purpose, we performed WGCNA for transcriptionally selected drought‐responsive genes using the R package WGNCA (Langfelder & Horvath, 2008). The automatic one‐step network construction and module detection method with the default settings were used, which included an unsigned type of topological overlap matrix (TOM). Briefly, we constructed the co‐expression networks using the absolute value of Pearson's correlation coefficient, which was raised to a power (softpower = 6) to create the adjacency matrix. The topological overlap distance calculated from the adjacency matrix is then clustered with the average linkage hierarchical clustering. Our modules were defined using the cutreeDynamic function with a minimum module size of 30 genes. A module eigengene distance threshold of 0.20 was used to merge highly similar modules use mergeCloseModules function. We set the edge threshold as 0.01 to determine biologically significant edges within each module. These parameters allowed for detection of a minimum number of large modules while visually respecting the pattern of correlation with the phenotypic gradients. Correlation analyses between module eigengenes and measured agronomic traits were applied to explore the biological significance of each module. A gene with the number of edges in the top 15% in a module was determined to be a hub gene.

## 3 RESULTS

### 3.1 Morphological differences between upland and lowland rice ecotypes

**FIGURE 1:**
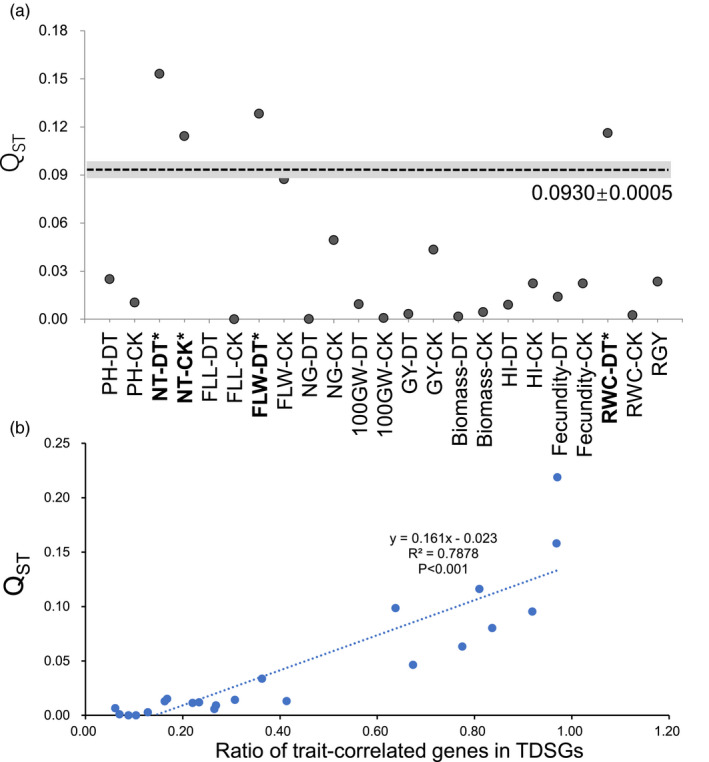
Morphological divergences between upland and lowland ecotypes and its association with transcriptomic divergence. (a) Comparisons between *Q*_ST_ values of measured traits and the neutral *F*_ST_ (0.0930 ± 0.0005, indicate by the dashed line and grey shallow). The trait in bold with “*” indicates its *Q*_ST_ is significantly higher than the neutral *F*_ST_. (b) Significant correlation between *Q*_ST_ of a measured trait and the ratio of the trait‐correlated genes in transcriptionally selected genes

**FIGURE 2:**
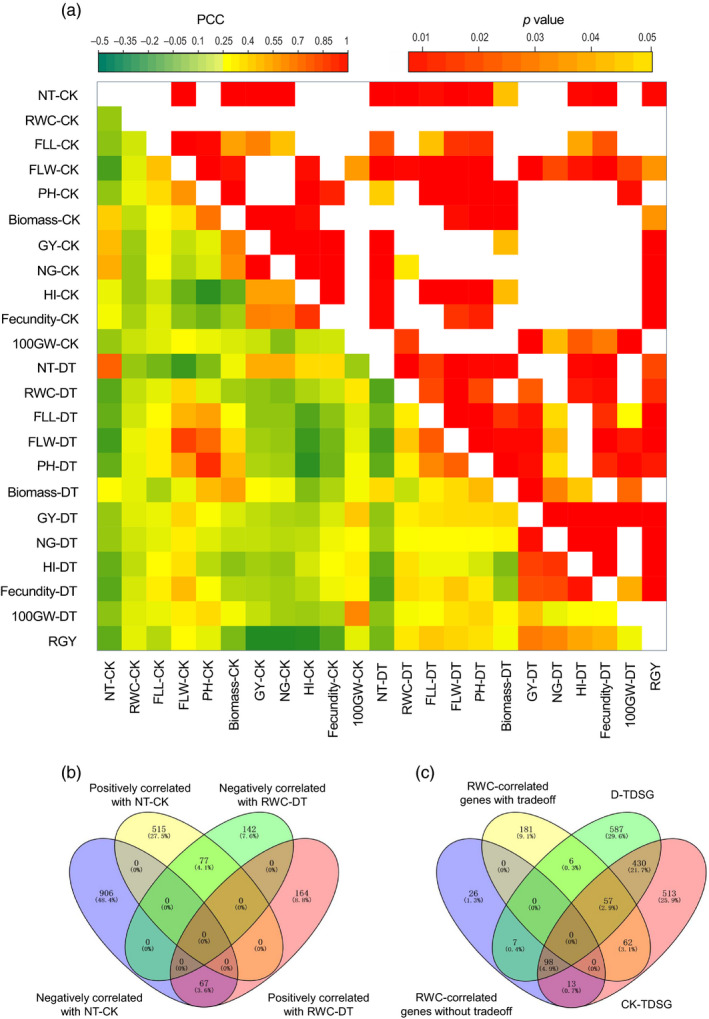
Morphological and transcriptomic trade‐offs between drought tolerance and productivity among typical genotypes. (a) Morphological trade‐offs revealed by correlations among agronomic and drought‐tolerant traits. Abbreviations: plant height (PH), number of tillers (NT), flag leaf length (FLL), flag leaf width (FLW), number of grains (NG), 100‐grain weight (100GY), grain yield (GY), harvest index (HI), relative water content (RWC), and relative grain yield (RGY). CK indicates a trait measured in the well‐watered field. DT indicates a trait measured in the drought‐treated field. (b) Venn diagram of RWC‐ and NT‐correlated genes. If a gene is negatively correlated with one trait but positively correlated with the other, it is defined as gene with potential trade‐offs. (c) Venn diagram of RWC‐correlated genes with trade‐offs or without trade‐offs in transcriptionally selected genes under DT (D‐TDSGs) and CK (CK‐TDSGs)

Drought had strong negative impacts on rice growth and reproduction, such as a significant decrease in PH, the NT, FLW, the NG, GY, biomass, the HI, and fecundity (Figure S3). The leaf water status, indicated by the RWC, became poorer under drought (Figure S3k). Upland and lowland rice exhibited significant differences in many agronomic traits. In the CK, lowland rice displayed more tillers, narrower flag leaves, more grains, a higher GY, more biomass, a higher HI, and better fecundity (Figure S3). In the DT, upland rice exhibited a greater PH, fewer tillers, and longer and wider flag leaves (Figure S3). Upland rice possessed better drought tolerance, as indicated by a greater RWC‐DT and RGY. The results were the same when the comparisons were between typical upland and lowland rice (Figure S4). The RGY and RWC‐DT were negatively correlated with many yield component traits in the CK, such as the NT, NG, GY, HI, and fecundity, among typical (Figure 2a) and total genotypes (Figure S5). This result indicated certain trade‐offs between drought tolerance and productivity in rice. Four measured traits (NT in both the CK and DT and FLW and RWC in the DT) had significantly higher *Q*_ST_ values than the neutral genomic *F*_ST_ calculated from intergenic SNPs (Figure 1a).

### 3.2 Genetic differentiation between upland and lowland rice ecotypes

**FIGURE 3:**
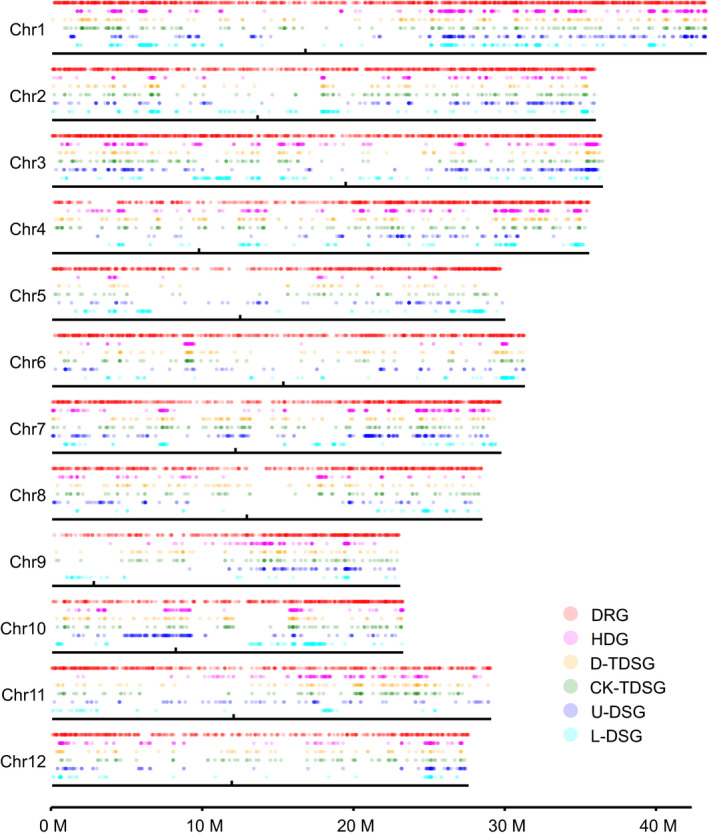
Distributions of drought‐responsive gene (DRG), highly differentiated genes (HDG), transcriptional selected genes under drought (D‐TDSG), transcriptional selected genes under well‐watered condition (CK‐TDSG), directionally selected genes in upland rice (U‐DSG), and directionally selected genes in lowland rice (L‐DSG) across the rice genome

Upland and lowland rice were generally separated along the first and second coordinates in the PCA based on their genomic SNPs (Figure S6a). This result was similar with the results from cluster analysis (Figure S7a) and STRUCTURE (Figure S7b), indicating a considerable level of genetic differentiation between the upland and lowland ecotypes. Based on these results, 40 and 50 typical upland and lowland rice genotypes were determined (Figure S7). By calculating the genetic divergence (*F_ST_*) for each gene, 2,708 genes (*F_ST_* > 0.592, beyond the 95% confidence interval (CI)) were determined to be HDGs between typical upland and lowland rice ecotypes. These genes were distributed unevenly across the genome based on single‐sample Kolmogorov–Smirnov test (*p* < .001) (Figure 3, Figure S8a). Based on GO enrichment, these HDGs were relevant to response to biotic stimulus (GO:0009607), response to external stimulus (GO:0009605), ncRNA catabolic process (GO:0034661), and rRNA catabolic process (GO:0016075), among others (Figure S9). However, no significant enrichment of genes in response to the eight environmental factors was detected in the HDGs (Table S7).

### 3.3 Expression variation under well‐watered and DT conditions

A total of 37,637 and 36,388 expressed genes were detected in samples from the CK and DT fields, respectively. Among these expressed genes, nearly one‐fourth (8,846) were identified as DRGs, which were evenly distributed across the genome (Figure 3, Figure S8b). The average coefficient of variation (C.V.) (Figure S10a) and expression diversity (*E_d_*) (Figure S10b) were significantly decreased in the DT based on Wilcoxon signed rank test. Furthermore, the C.V. and *E_d_* in upland rice were significantly higher than that in lowland rice under both CK and DT conditions based on Wilcoxon signed rank test (Figure S10).

### 3.4 Transcriptomic divergence under well‐watered and DT conditions

In total, 1,185 and 1,173 genes were determined to be TDSGs with expression under directional selection in the CK and DT fields, respectively (Figure 3, Figure S8e). The SI of the TDSGs was positive under both the CK (mean = 0.943, 95% CI from 0.939 to 0.948) and DT (mean = 0.915, 95% CI from 0.909 to 0.921), which was consistent with the results by the rank‐based method. CK‐TDSGs and D‐TDSGs were distributed frequently within the same regions (Figure S8d) and shared 585 (33.0%) common genes (Figure S8e). The mean *F_ST_* values of the CK‐TDSGs (0.383 ± 0.007) and DT‐TDSGs (0.421 ± 0.006) were significantly higher than the genomic average (0.261 ± 0.001) based on both Mann–Whitney (*p* < .001) and Kolmogorov–Smirnov (*p* < .001) tests (Figure S8f). This result indicated that the genes were preferential in sequence‐based selection.

Directional selection increased gene expression divergence (*Q*_ST_ of gene expression) (Figure S11a) and *E_d_* under both CK and DT conditions (Figure S11b). Upland and lowland ecotypes were not clearly separated by PCA under either the CK or DT condition based on the FPKMs of total expressed genes (Figure S12), while typical upland and lowland landraces were generally separated along the second coordinate when the FPKMs of TDSGs were used (Figure S6b and c). The CK‐TDSGs were related to maturation of SSU‐rRNA, maturation of SSU‐rRNA, chloroplast localization, plastid localization, photosystem II assembly, among others (Figure S13). The D‐TDSGs were related to isopentenyl diphosphate biosynthetic process, phospholipid biosynthetic process, aromatic amino acid family metabolic process, and glyceraldehyde‐3−phosphate metabolic process, among others (Figure S14). Noticeably, the drought‐ and cold‐responsive genes were significantly enriched by both CK‐TDSGs and D‐TDSGs (Table S8), indicating that transcriptomic divergence played a role in rice adaptation to drought and cold.

### 3.5 Transcriptomic divergence contributed to morphological divergence and drought adaptation

Among the 155 functionally characterized drought‐tolerant genes and 101 NT‐related genes (Table S9), only seven drought‐resistant and three NT‐related genes were transcriptionally selected under either condition or both conditions (Table S10). Moreover, the expression patterns of only *OsAKT1* and *OsGL1‐8* matched the observed higher drought tolerance in upland rice (Table S10). This result indicated that morphological differences in drought tolerance and NT between the two rice ecotypes could not simply be explained by expression divergences between the abovementioned genes.

To find a potential transcriptomic explanation for the morphological divergences between the ecotypes, we further calculated the enrichment ratio of trait‐correlated genes in the TDSGs. There were always thousands of genes significantly correlated (*p* < .05) with morphological traits measured in CK or DT fields (Table S11). The *Q*_ST_ of a trait was significantly correlated with the ratio of trait‐correlated genes in TDSGs (Figure 1b) among typical genotypes. This result indicated that the transcriptomic divergence between the two ecotypes contributed to their morphological divergence.

RWC‐D was a key physiological trait for estimating drought tolerance, which was divergently selected between the two rice ecotypes based on the *Q*_ST_‐*F*_ST_ comparison (Figure 1a). Based on Fisher's exact test, if a drought‐responsive D‐TDSG was negatively correlated with the RWC, its expression level tended to be lower in upland rice. In contrast, if a drought‐responsive TDSG was positively correlated with the RWC, its expression level tended to be higher in upland rice (Table S12). This result indicated that expressions of RWC‐related D‐TDSGs were beneficial to maintain a better water status in upland rice, which can promote its drought adaptation.

NT is an important agronomic trait that greatly affects rice yield potential. It was divergently selected between the two rice ecotypes based on the *Q*_ST_‐*F*_ST_ comparison (Figure 1a). There were 144 genes with opposite correlations with RWC‐DT and NT‐CK (positively correlated with one trait but negatively correlated with the other) (Figure 2b), indicating transcriptional trade‐offs between rice drought tolerance and tillering ability. A significantly larger proportion of these genes (81.9%, *p* value of Fisher's exact test < .001) than of other RWC‐correlated genes (40.8%) were directionally selected in the CK and DT fields (Figure 2c). This difference could partially explain the observed trade‐offs between RWC and NT.

### 3.6 Sequence‐based directional selection in upland and lowland rice

Expression divergence (*Q*_ST_ of gene expression) was significantly correlated with genetic divergence (*F_ST_* of gene expression) (Figure S15a). Furthermore, gene expression variation (C.V.) (Figure S15b) and expression diversity (*E_d_*) (Figure S15c) were significantly correlated with the genetic diversity of the gene. To identify the underlying genetic mechanism, we further scanned for signs of recent directional selection on gene sequences. We detected 1,669 and 1,655 genes with signs of recent directional selection in upland (U‐DSG) and lowland (L‐DSG) rice, respectively (Figure 3, Figure S8e). These genes rarely overlapped (Figure S8e) and were distributed separately in the rice genome (Figure S8c), indicating that the domestication of upland and lowland rice involved divergent groups of genes. Moreover, the U‐DSGs (0.475 ± 0.007) and L‐DSGs (0.491 ± 0.006) both had significantly higher *F_ST_* values (*p* < .001 based on both Mann–Whitney and Kolmogorov–Smirnov tests) than the genomic average (Figure S8f). It was noteworthy that genes in responses to drought and alkali were enriched in the U‐DSGs (Table S7). The U‐DSGs (cellular transition metal ion homeostasis, isoleucine metabolic process, immune effector process, RNA methylation, Notch signaling pathway, etc.) (Figure S16) and L‐DSGs (organic substance metabolic process, primary metabolic process, amide biosynthetic process, lipid metabolic process, etc.) (Figure S17) were related to different biological processes.

### 3.7 *cis*‐element‐altering SNPs have impacts on gene expression and contribute to gene expression divergence

To test the impacts of the transcriptional regulatory region on transcriptional selection, we scanned transcription‐regulating regions (upstream −2,000 to the transcription start site) for *cis*‐element‐altering SNPs. A total of 17,263 genes (52.3% of total analyzed genes) across the rice genome were found to contain *cis*‐element‐altering SNPs. Meanwhile, 759 CK‐TDSGs (64.0%) and 814 D‐TDSGs (69.4%) contained *cis*‐element‐altering SNPs. The two proportions were significantly higher than that in total genes based on Fisher's exact test (*p* < .001 for both tests). In addition, highly differentiated TDSGs (284 out of 386, 73.6%) contained significantly higher proportions of *cis*‐element‐altering genes than neutral TDSGs (854 out of 1,387, 61.5%) did, based on Fisher's exact test (*p* = .047). These results suggested that *cis*‐element‐altering SNPs should contribute to gene expression divergence between rice ecotypes. After testing with ten highly differentiated TDSGs with *cis*‐element‐altering SNPs, we found that the expression divergences between haplotypes were generally greater than divergences between ecotypes for three genes (Table S11). These results indicated that mutations in *cis*‐elements impacted gene expression.

### 3.8 The gene co‐expression network contributed to rice transcriptomic adaptation to drought

**FIGURE 4:**
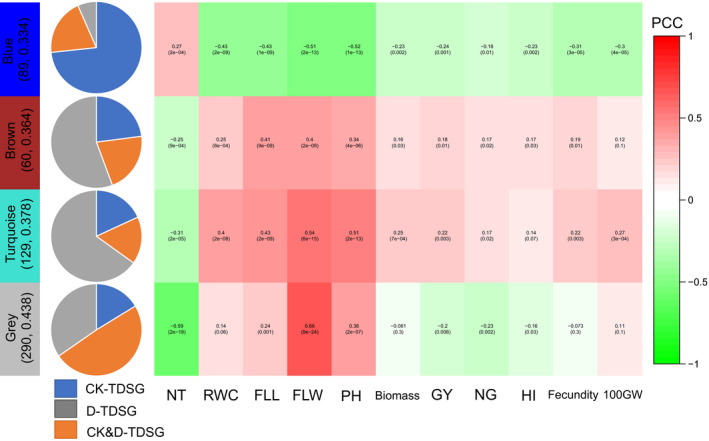
Compositions of four modules in the weighted gene co‐expression network and correlations (Pearson correlation coefficient, PCC) between their eigengenes and the measured traits. The number in the bracket indicates number of nodes in the module and the averaged *F*_ST_. The pie chart describes the proportion of transcriptional selected genes under drought (D‐TDSG) and under well‐watered condition (CK‐TDSG) in the module. The number in the bracket in the PCC matrix indicates *p* value from correlation analysis

We conducted WGCNA using 612 drought‐responsive TDSGs to test the contribution of the co‐expression network to rice drought adaptation. Four biologically significant modules were obtained that were significantly corrected with RWC. This result indicated that the co‐expression network here was associated with drought resistance. It was also noteworthy that the correlations of module eigengenes with NT and RWC were always opposed, providing additional transcriptomic explanations for their observed trade‐offs (Figure 4). The co‐expression network contained 147 HDGs (25.9% of the total TDSGs), which could promote the transcriptomic adaptation of upland rice to the drought‐prone environment *via* a co‐expression network. Meanwhile, 78.2% (115 out of 147) HDGs contained *cis*‐element‐altering SNPs. Therefore, we considered that eleven highly differentiated hub genes with *cis*‐element‐altering SNPs in the gene co‐expression network should play important roles in rice transcriptomic adaptation to drought (Table 1, Table S12).

**TABLE 1:** Information of 11 highly differentiated hub genes with *cis*‐element‐altering SNPs in the gene co‐expression network

Gene ID	*F*_ST_	Module	Correlated traits in CK (FDR < 0.05)	Correlated traits in DT (FDR < 0.05)
Os01g0209800	0.664	blue	FLW, NT, PH, NG, GY, HI	FLL, FLW, NT, PH, fecundity
Os10g0442100	0.902	blue	FLL, FLW, NT, PH, HI, 100GW, fecundity	FLL, FLW, NT, PH, RWC, GY, NG, HI, fecundity
Os01g0628700	0.699	brown	FLL, FLW, NT, PH	FLL, FLW, NT, PH, fecundity
Os01g0908700	0.698	brown	FLL, FLW	FLL, FLW, NT, PH
Os12g0594600	0.901	grey	FLW, NT, PH	FLL, FLW, NT, PH, RWC, fecundity
Os02g0217800	0.641	grey	FLW, NT, PH, NG, GY, HI	FLL, FLW, NT, PH, RWC
Os11g0485200	0.608	grey	FLW, NT	FLL, FLW, NT, PH, RWC, GY, fecundity
Os03g0382350	0.694	grey	FLL, FLW, NT, PH, HI, 100GW, fecundity	FLL, FLW, NT, PH, RWC, 100GW, fecundity
Os02g0218400	0.674	grey	FLW, NT, PH, NG, GY, HI, fecundity	FLL, FLW, NT, PH, RWC, 100GW
Os01g0130900	0.626	turquoise	FLW, NT	FLW, RWC
Os01g0692700	0.602	turquoise	FLL, FLW, NT, PH	FLL, FLW, NT, PH, fecundity

Abbreviations: 100GW, 100‐grain weight;CK, well‐watered; DT, drought‐treated; FLL, flag leaf length; FLW, flag leaf width; GY, grain yield; HI, harvest index; NG, number of grains; NT, number of tillers; PH, plant height; RGY, relative grain yield; RWC, relative water content.

## 4 DISCUSSION

### 4.1 Transcriptomic divergence contributes to the adaptation of upland rice to drought‐prone upland environments

Variation in gene expression plays an essential role in plant adaptation to changing environments (Dayan et al., 2015; Xu et al., 2015, 2016). Greater variation in gene expression could indicate higher evolutionary potential (Pavey et al., 2010; Xu et al., 2015). In this study, greater variation in gene expression was detected in upland rice than in lowland rice under both DT and well‐watered conditions, suggesting that the transcriptome of upland rice may be more flexible in drought‐prone environments.

In many previous studies, the formation of an adaptive trait in a population was attributed to expression divergence of several or a group of genes (Aikawa, Kobayashi, Satake, Shimizu, & Kudoh, 2010; Dayan et al., 2015; Gould et al., 2018). We observed improved drought resistance in upland rice, which is consistent with the results of many previous studies (Lyu et al., 2014; Xia et al., 2019). This result could not be explained by the characterized drought‐tolerant genes, as only limited expression divergences were recorded. In contrast, we detected great transcriptomic divergence between upland and lowland rice ecotypes, which contained thousands of TDSGs. We found that genes responding to drought were significantly enriched in the TDSGs. Transcriptional selection contributes greatly to morphological divergence between rice ecotypes and could promote drought adaptation in upland rice by maintaining a better water status. In summary, the directional selection on the transcriptome during rice domestication and its consequent transcriptomic divergences play an important role in the adaptation of upland rice to the drought‐prone upland environment. However, our results are gained merely from leaf tissues, which could only reflect aboveground drought tolerance. The transcriptomic divergence, which leads to differences in the root develop and architecture, a vital part of drought avoidance, requires further investigation.

### 4.2 Promotion of rice transcriptomic adaptation to drought by key HDGs in gene co‐expression network

Previous studies have demonstrated that plant adaptation may more commonly proceed *via* regulatory rather than structural (i.e., protein‐coding) changes because regulatory mutations have spatially or temporally circumscribed effects (Gould et al., 2018; Guo et al., 2016). Based on our data, mutations in *cis*‐elements have impacts on gene expression and cause expression divergences. However, we also note that epigenetic mechanisms, which require further investigation, could play roles in transcriptomic divergences between upland and lowland rice (Xia et al., 2016).

Plant ecological adaptation to environmental factors commonly involves limited genomic regions and/or genes (Nosil, Funk, & Ortiz‐Barrientos, 2009). The genetic divergence between upland and lowland rice exhibited such a pattern (Lyu et al., 2014; Xia et al., 2019). However, transcriptomic divergences between ecologically differentiated populations involve many more genes (Dayan et al., 2015; Gould et al., 2018). For instance, only 17.8% of the TDSGs were genetically differentiated between the two rice ecotypes. Thus, the remaining ~ 80% of TDSGs were the result of direct or indirect interactions with those HDGs. Based on the WGCNA in this study, we found all the four modules, which contained several highly differentiated hub genes, were associated with drought tolerance. For this reason, we consider the hub HDGs, particularly ones containing *cis*‐element‐altering SNPs, to promote the transcriptomic adaptation of upland rice to drought *via* the co‐expression network.

### 4.3 Transcriptomic trade‐off between drought tolerance and productivity

The trade‐off between productivity and drought tolerance in rice has been widely discussed at the gene (Jung, Lee, Choi, & Kim, 2015; Zhang, Liu, et al., 2016) and genome scales (Vikram et al., 2015; Xia et al., 2019). In this study, we detected a strong trade‐off between drought tolerance and tillering ability at the transcriptome scale. The transcriptomic trade‐off was also reflected by the opposite correlations of module eigengenes with RWC‐DT and number of tillers. The transcriptomic trade‐off may pose some difficulties in the efficient utilization of drought‐resistant genes by constitutive overexpression (Jung et al., 2015; Zhang, Liu, et al., 2016). In contrast, stress‐induced expression of drought‐tolerant genes can be beneficial under drought without penalties under normal conditions (Reis et al., 2014; Xiao, Huang, Tang, & Xiong, 2007). That is why mutations in *cis*‐elements, which can conditionally regulate gene expression, accumulated in highly differentiated TDSGs.

### 4.4 Agricultural implications of breeding drought‐tolerant cultivars

Although many drought‐tolerant genes exhibit good performance under laboratory conditions, their success in commercial fields is rare (Fang & Xiong, 2015). This discrepancy occurs because plant adaptation to drought is a systematic response from the cell to the whole plant (Farooq, Wahid, Lee, Ito, & Siddique, 2009). A drought‐tolerant gene with a minor effect may not be positively selected during upland–lowland rice differentiation. In contrast, the key genes, which promote rice transcriptomic adaptations to drought, can have systematic effects on drought resistance. They are thus valuable candidates for improving drought tolerance in rice breeding.

## 5 DATA ARCHIVING AND AVAILABILITY STATEMENT

The raw data of RNA‐seq and resequencing are available at the NCBI Sequence Read Archive (SRA) (PRJNA306542) and (PRJNA260762), respectively. The phenotype data are available in “Xia et al., 2019 (https://doi.org/10.1016/j.molp.2018.12.011)” as “Table S9”.

## CONFLICT OF INTEREST

The authors declare no competing financial interests.

## AUTHOR CONTRIBUTIONS

H.X. and L.J.L. designed the experiments. Z.L., J.X., H.X., and M.G. conducted the field and molecular experiments. H.X., Z.L., X.S.M., and L.W. analyzed the data. G.L.L. and X.Q.Y. prepared plant materials used in this study. H.X., Z.L., and L.J.L. wrote the manuscript.

## Supporting information

Fig S1‐S17

Table S1‐S14
