# Supplementary material for: Transcriptomic divergence between upland and lowland ecotypes contributes to rice adaptation to a drought‐prone agroecosystem
Source: Evol Appl. 2020 Jul 16;13(9):2484–96. doi: 10.1111/eva.13054 (PMC7513727; doi:10.1111/eva.13054)
Supplement: Supplementary file 1 — Fig S1‐S17 [file EVA-13-2484-s001.pdf]

Supplementary Figure legends

Figure S1. Validation of expression levels of six drought-resistance genes calculated from RNA-sequencing by qPCR.

Figure S2. Validation of three cis-element altering SNPs called from resequencing at Os06g0269200 by Sanger method.

Figure S3. Performances of typical upland (dark grey) and lowland (light grey) rice genotypes under drought-treated (left columns in straight line) and well-watered (right columns in dashed line) fields.

Figure S4. Performances of total upland (dark grey) and lowland (light grey) rice genotypes under drought-treated (left columns in straight line) and well-watered (right columns in dashed line) fields.

Figure S5. Morphological tradeoffs revealed by correlations between agronomic and drought-tolerant traits among total genotypes. Abbreviations: plant height (PH), number of tillers (NT), flag leaf length (FLL), flag leaf width (FLW), number of grains (NG), 100-grain weight (100GY), grain yield (GY), harvest index (HI), relative water content (RWC), and relative grain yield (RGY). CK indicates a trait measured in the well-watered field. DT indicates a trait measured in the drought-treated field.

Figure S6. Results of principal coordinate analysis (PCA) based on genome-wide SNPs (a) and expression levels of transcriptionally selected genes in CK (b) and DT (c).

Figure S7. Population structure of 124 rice genotypes inferred by the N-J phylogenetic tree (a) and structure (b).

Figure S8. Distribution of highly differentiate genes (HDGs) (a), drought-responsive genes (DRGs) (b), sequence-based selective genes (DSGs) in upland and lowland rice (c), and transcriptionally selected genes (TDSGs) in CK and DT conditions (d), as well as their Venn diagram (e) and genetic divergence (f).

Figure S9. Top 15 (by p value) GO terms of biological process enriched in highly differentiated genes.

Figure S10. Coefficient of variance (C.V.) (a) and expression diversity (b) in total, upland, and lowland genotypes under drought (black column) and well-watered (light grey column) conditions.

Figure S11. Comparisons of Q_{ST} (a) and expression diversity (b) between total genes and transcriptionally selected genes (TDSGs) under drought and well-watered conditions, respectively.

Figure S12. Results of principal coordinate analysis based on total transcriptomic data from typical genotypes.

Figure S13. Top 15 (by p value) GO terms of biological process enriched in transcriptionally selected genes under the well-watered condition.

Figure S14. Top 15 (by p value) GO terms of biological process enriched in transcriptionally selected genes under the drought condition.

Figure S15. Genetic bases of variation in gene expression and expression divergence. (a) Correlation between genetic divergence (F_{ST}) and expression divergence (Q_{ST}). (b) Correlation between genetic diversity and coefficient of variance of gene expression. (c) Correlation between genetic diversity and expression diversity.

Figure S16. Top 15 (by p value) GO terms of biological process enriched in directionally selected genes in upland rice.

Figure S17. Top 15 (by p value) GO terms of biological process enriched in directionally selected genes in lowland rice.

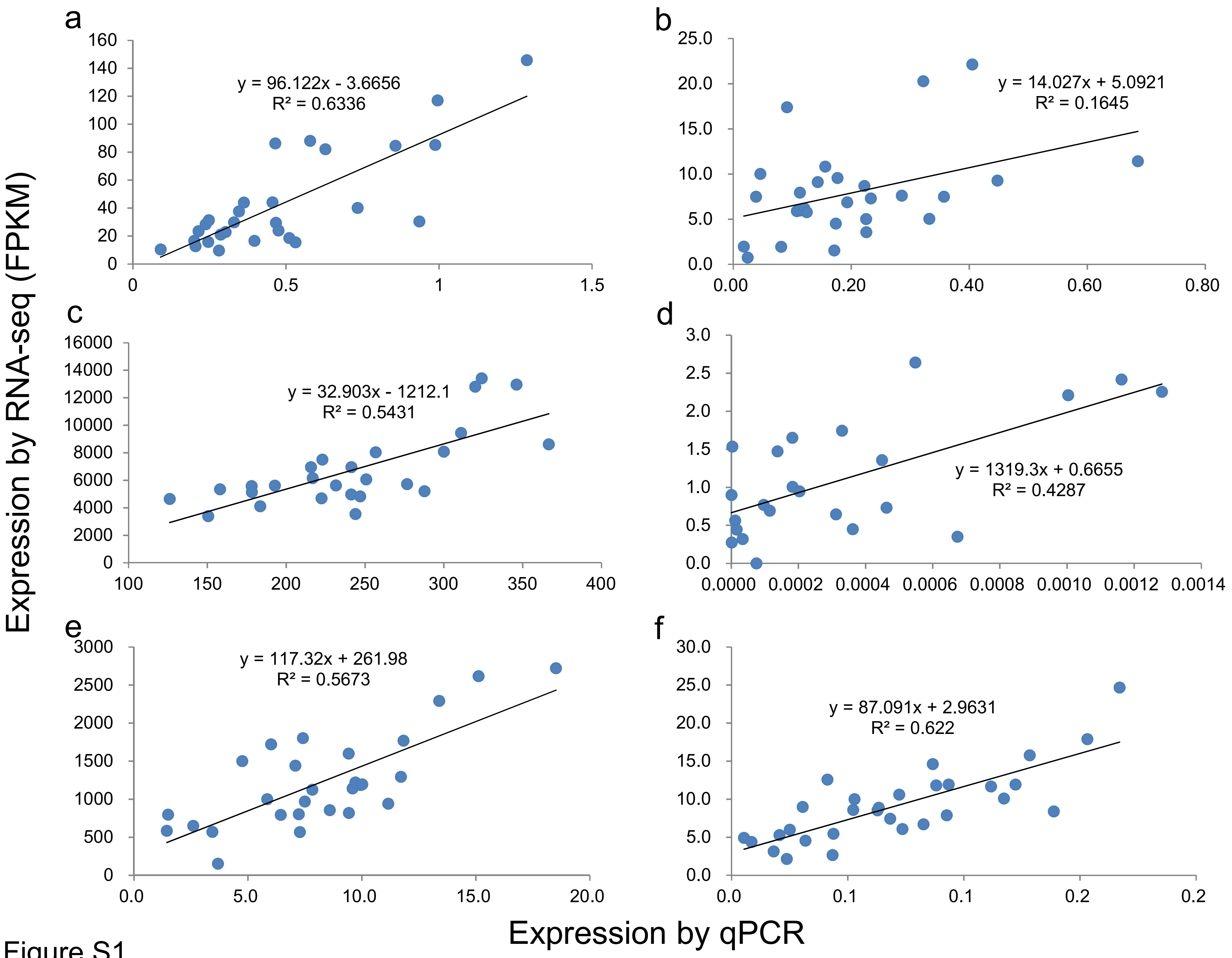

a
y = 96.122x - 3.6656
R² = 0.6336
b
y = 14.027x + 5.0921
R² = 0.1645
c
y = 32.903x - 1212.1
R² = 0.5431
d
y = 1319.3x + 0.6655
R² = 0.4287
e
y = 117.32x + 261.98
R² = 0.5673
f
y = 87.091x + 2.9631
R² = 0.622
Expression by RNA-seq (FPKM)
Expression by qPCR

Figure S1

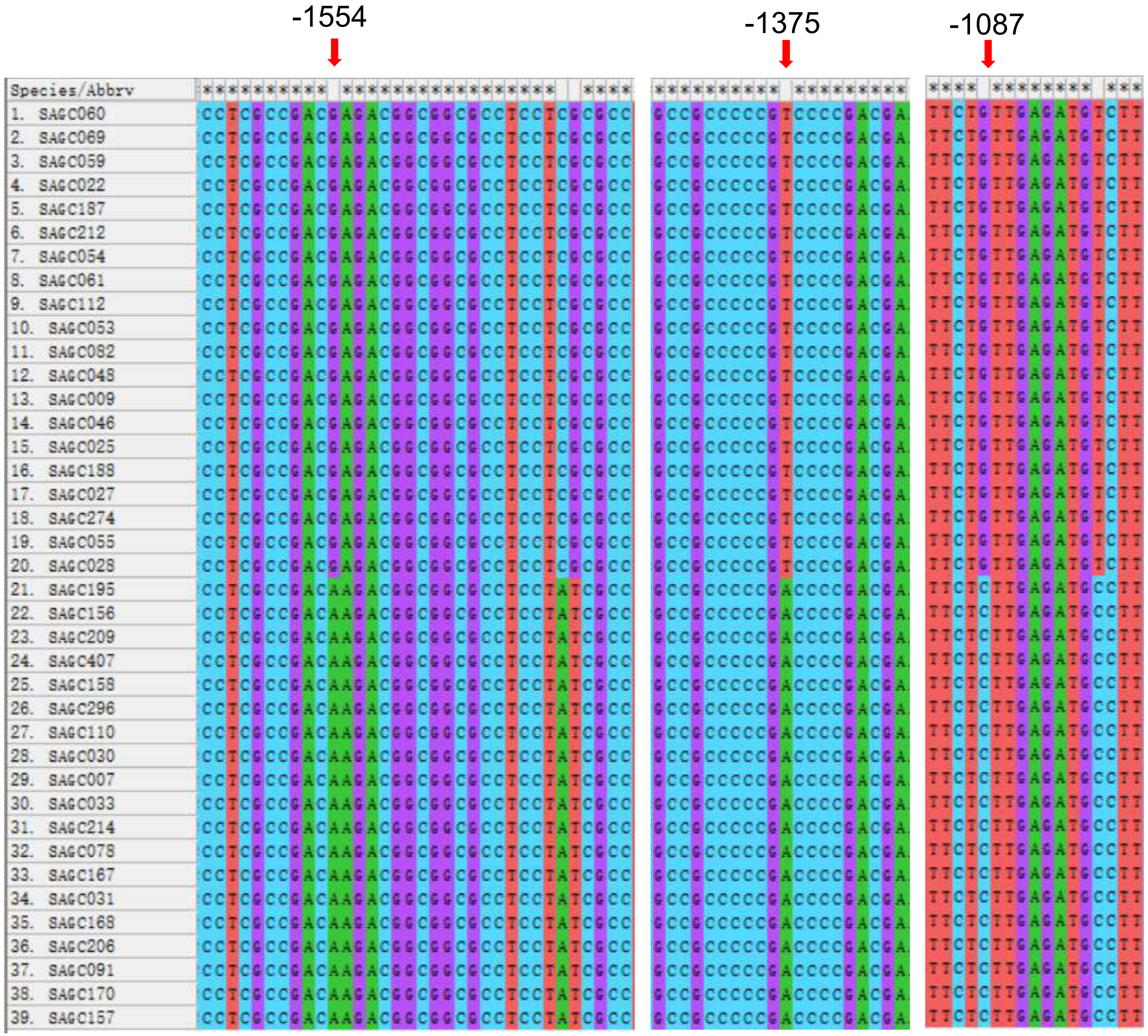

Genotype	-1554	-1375	-1087
SAGC060	G	T	G
SAGC069	G	T	G
SAGC059	G	T	G
SAGC022	G	T	G
SAGC187	G	T	G
SAGC212	G	T	G
SAGC054	G	T	G
SAGC061	G	T	G
SAGC112	G	T	G
SAGC053	G	T	G
SAGC082	G	T	G
SAGC048	G	T	G
SAGC009	G	T	G
SAGC046	G	T	G
SAGC025	G	T	G
SAGC188	G	T	G
SAGC027	G	T	G
SAGC274	G	T	G
SAGC055	G	T	G
SAGC028	G	T	G
SAGC195	-	A	C
SAGC156	-	A	C
SAGC209	-	A	-
SAGC407	A	A	C
SAGC158	-	A	C
SAGC296	-	A	C
SAGC110	-	A	C
SAGC030	A	A	S
SAGC033	-	A	C
SAGC007	A	A	C
SAGC214	-	A	C
SAGC078	-	A	C
SAGC167	A	A	C
SAGC031	-	A	C
SAGC168	-	A	C
SAGC206	-	A	-
SAGC091	-	A	-
SAGC170	A	A	C
SAGC157	-	A	C

Figure S2

Cis-element altering SNP generated by Sanger sequencing

Cis-element altering SNP generated by resequencing

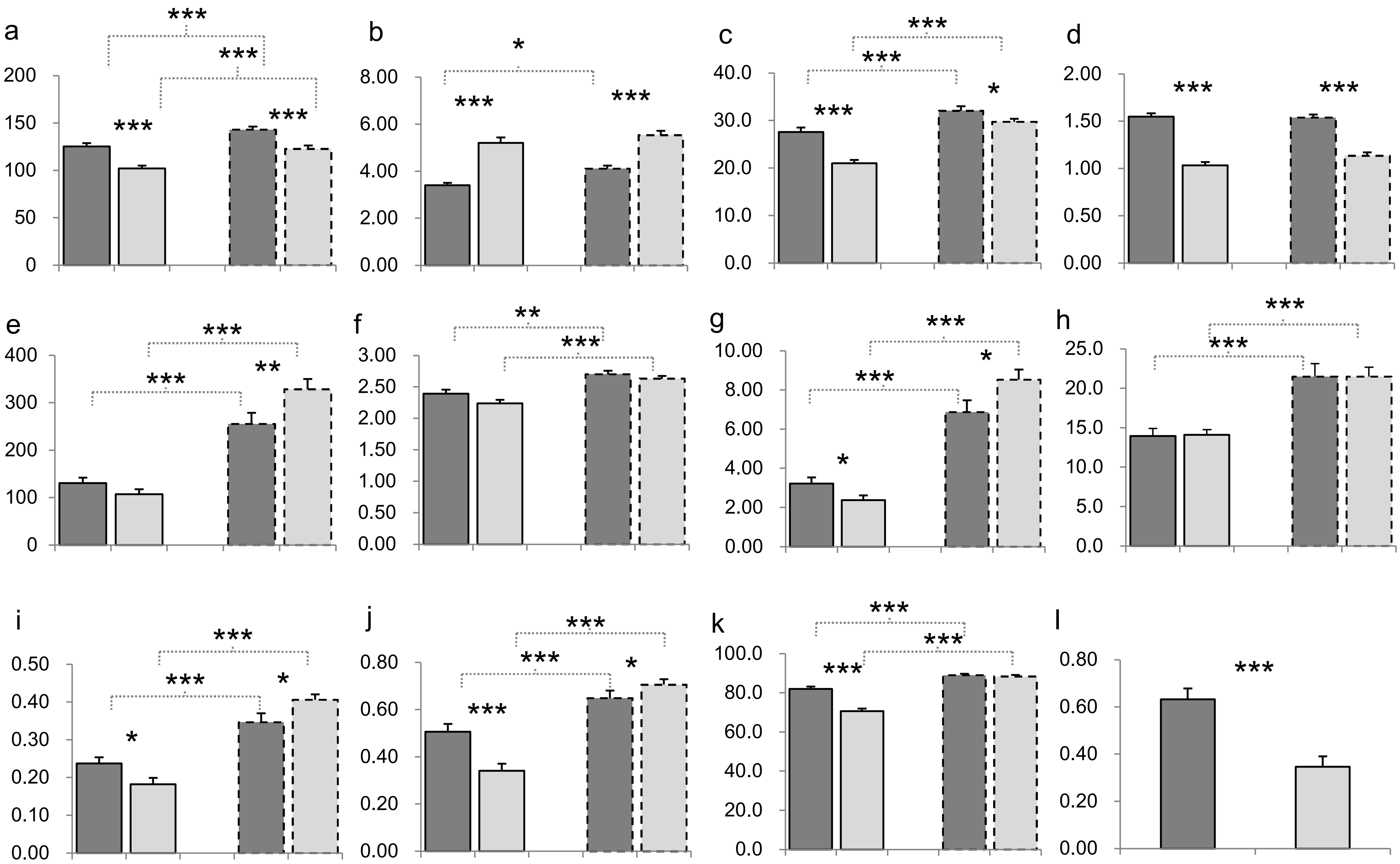
a
0 50 100 150 200
b
0.00 2.00 4.00 6.00 8.00
c
0.0 10.0 20.0 30.0 40.0
d
0.00 0.50 1.00 1.50 2.00
e
0 100 200 300 400
f
0.00 0.50 1.00 1.50 2.00 2.50 3.00
g
0.00 2.00 4.00 6.00 8.00 10.00
h
0.0 5.0 10.0 15.0 20.0 25.0
i
0.00 0.10 0.20 0.30 0.40 0.50
j
0.00 0.20 0.40 0.60 0.80
k
0.0 20.0 40.0 60.0 80.0 100.0
l
0.00 0.20 0.40 0.60 0.80
*
**
***

Figure S3

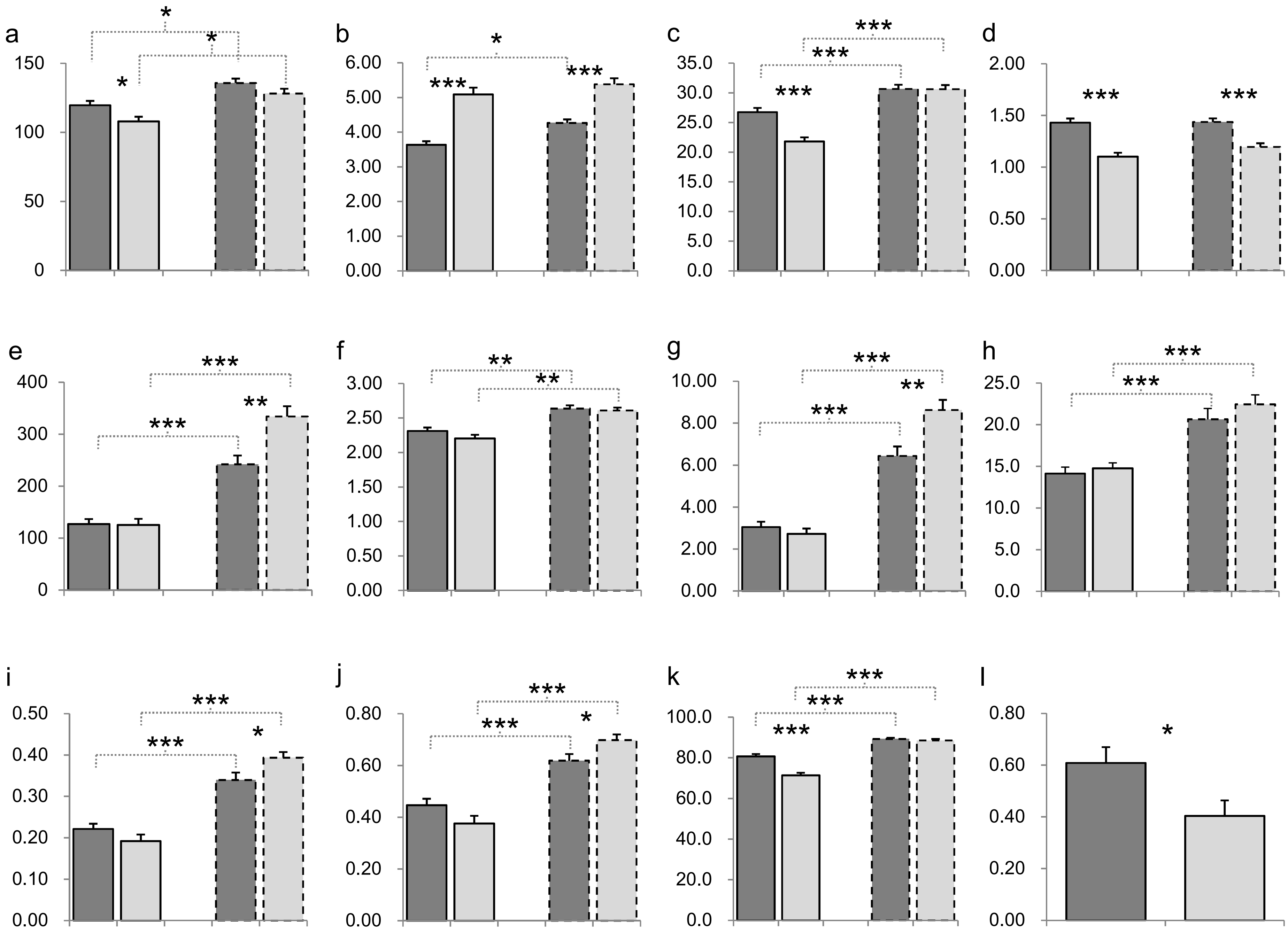

Figure S4

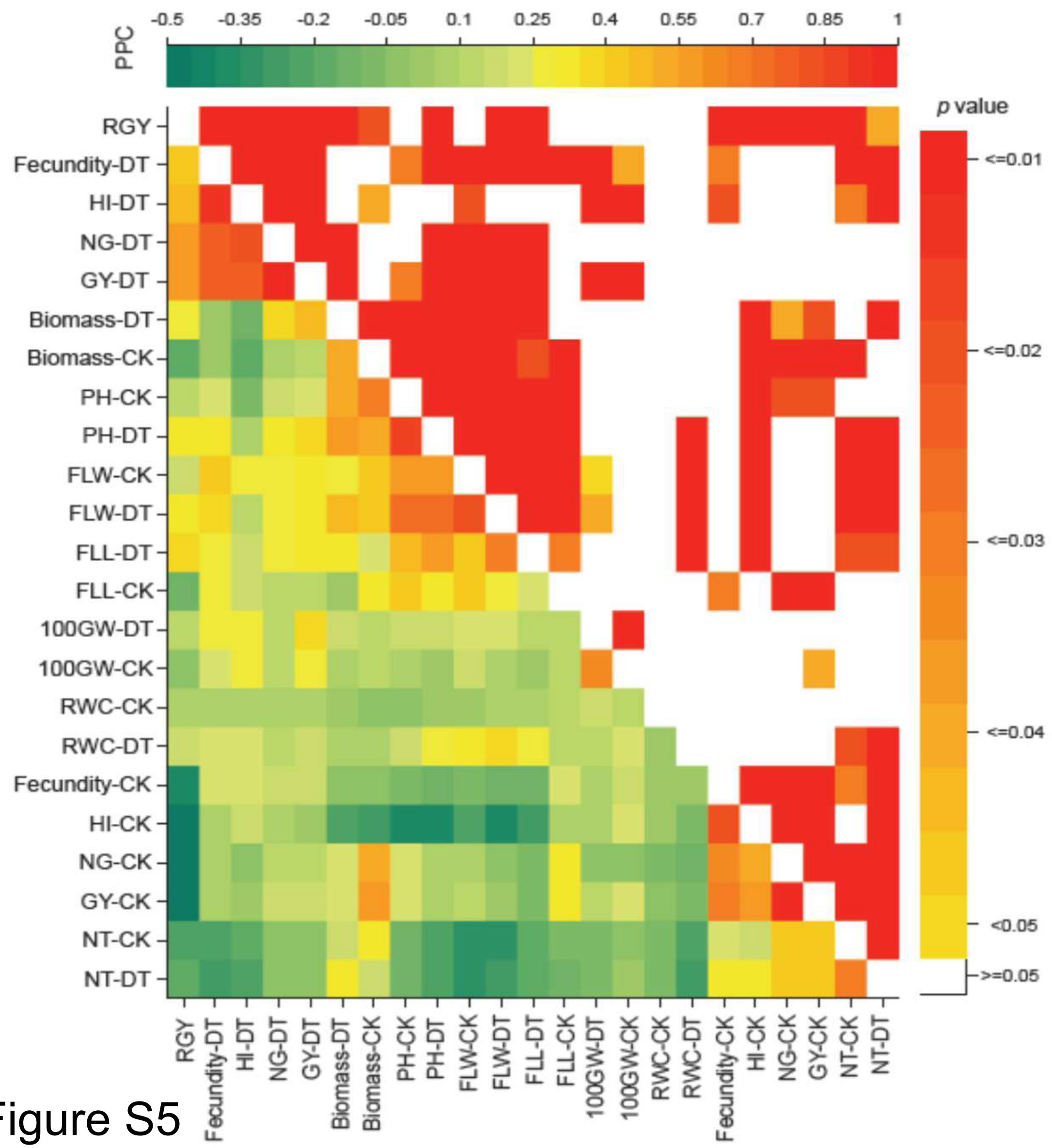
PPC
-0.5
-0.35
-0.2
-0.05
0.1
0.25
0.4
0.55
0.7
0.85
1
p value
<=0.01
<=0.02
<=0.03
<=0.04
<0.05
>=0.05
RGY
Fecundity-DT
HI-DT
NG-DT
GY-DT
Biomass-DT
Biomass-CK
PH-CK
PH-DT
FLW-CK
FLW-DT
FLL-DT
FLL-CK
100GW-DT
100GW-CK
RWC-CK
RWC-DT
Fecundity-CK
HI-CK
NG-CK
GY-CK
NT-CK
NT-DT

Figure S5

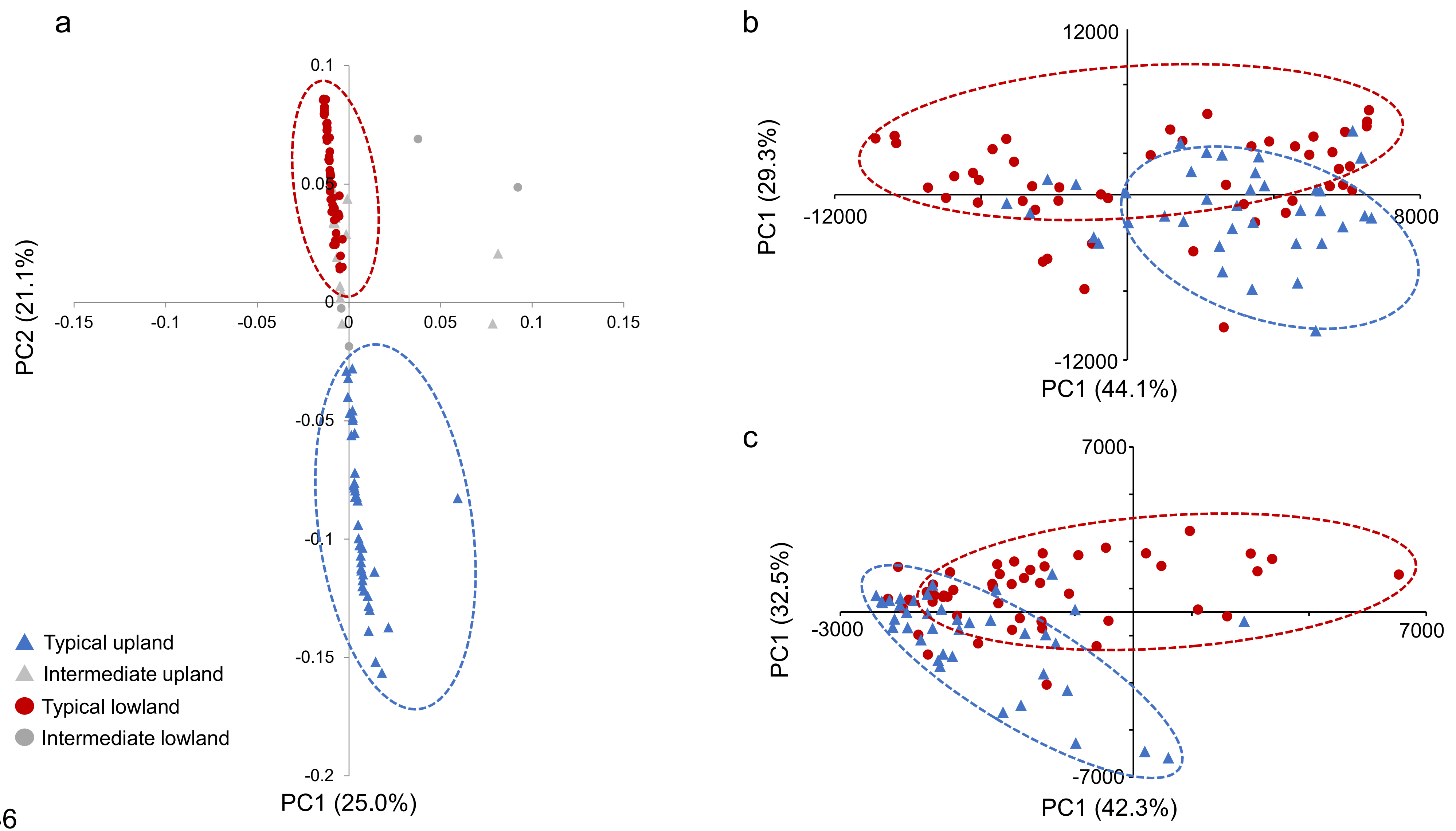

a
0.1
0.05
0
-0.05
-0.1
-0.15
-0.2
-0.15
-0.1
-0.05
0
0.05
0.1
0.15
PC2 (21.1%)
PC1 (25.0%)
Typical upland
Intermediate upland
Typical lowland
Intermediate lowland
b
12000
-12000
8000
-12000
PC1 (29.3%)
PC1 (44.1%)
c
7000
-3000
7000
-7000
PC1 (32.5%)
PC1 (42.3%)

Figure S6

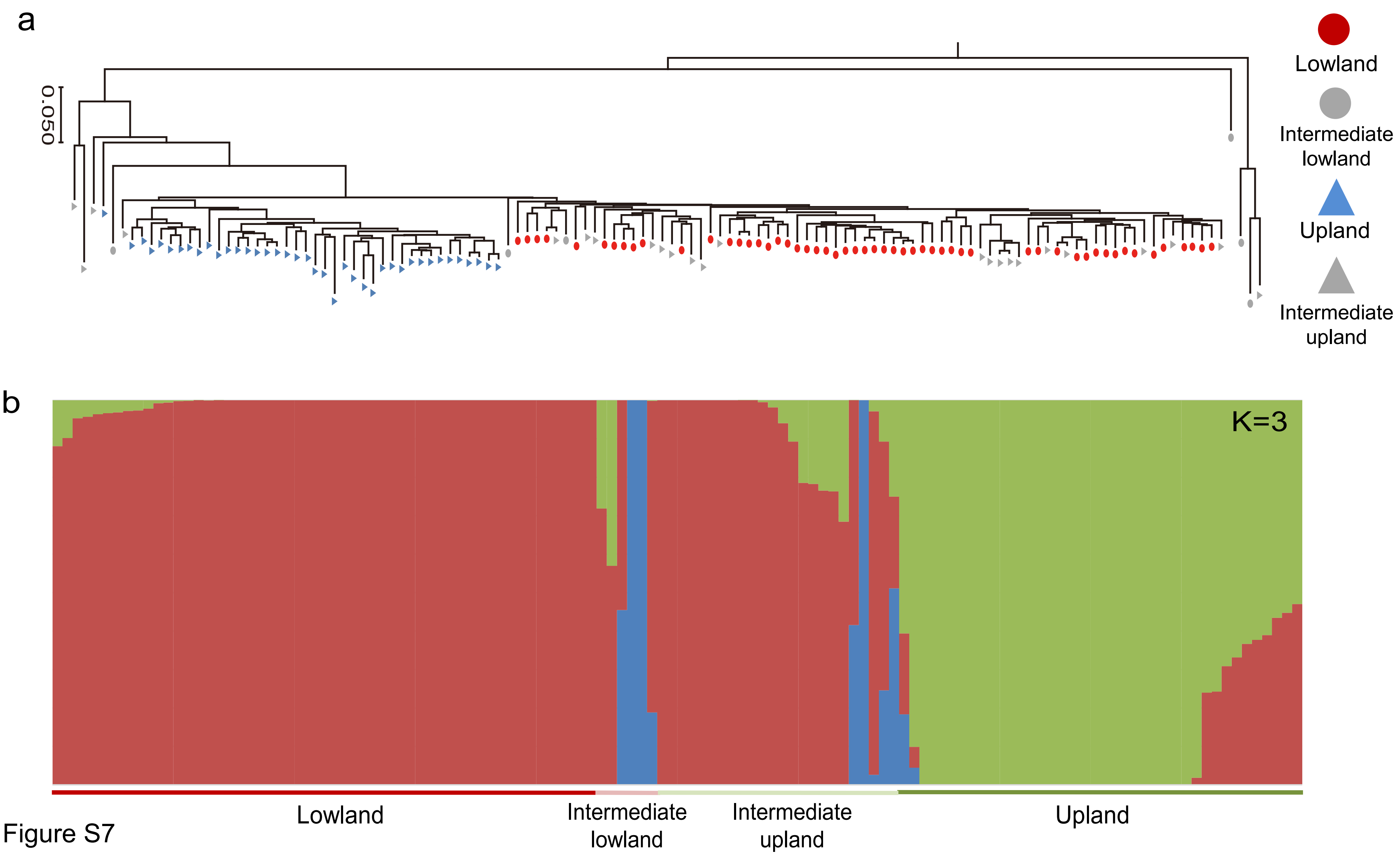
a
0.050
Lowland
Intermediate lowland
Upland
Intermediate upland
b
K=3
Lowland
Intermediate lowland
Intermediate upland
Upland

Figure S7

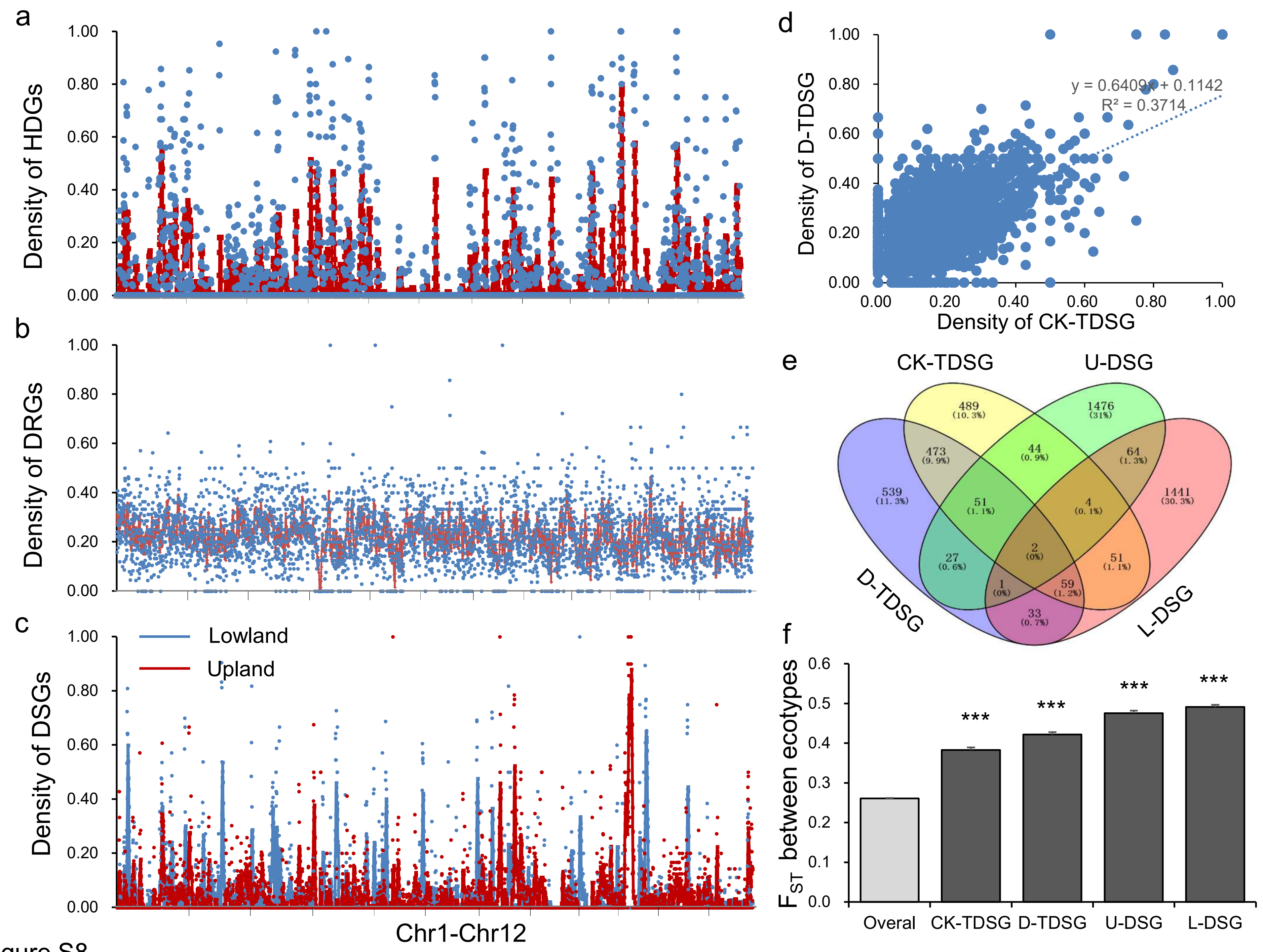
a
Density of HDGs
b
Density of DRGs
c
Density of DSGs
Lowland
Upland
Chr1-Chr12
d
Density of D-TDSG
Density of CK-TDSG
y = 0.6409x + 0.1142
R² = 0.3714
e
CK-TDSG
U-DSG
D-TDSG
L-DSG
f
F_ST between ecotypes
Overal
CK-TDSG
D-TDSG
U-DSG
L-DSG

Figure S8

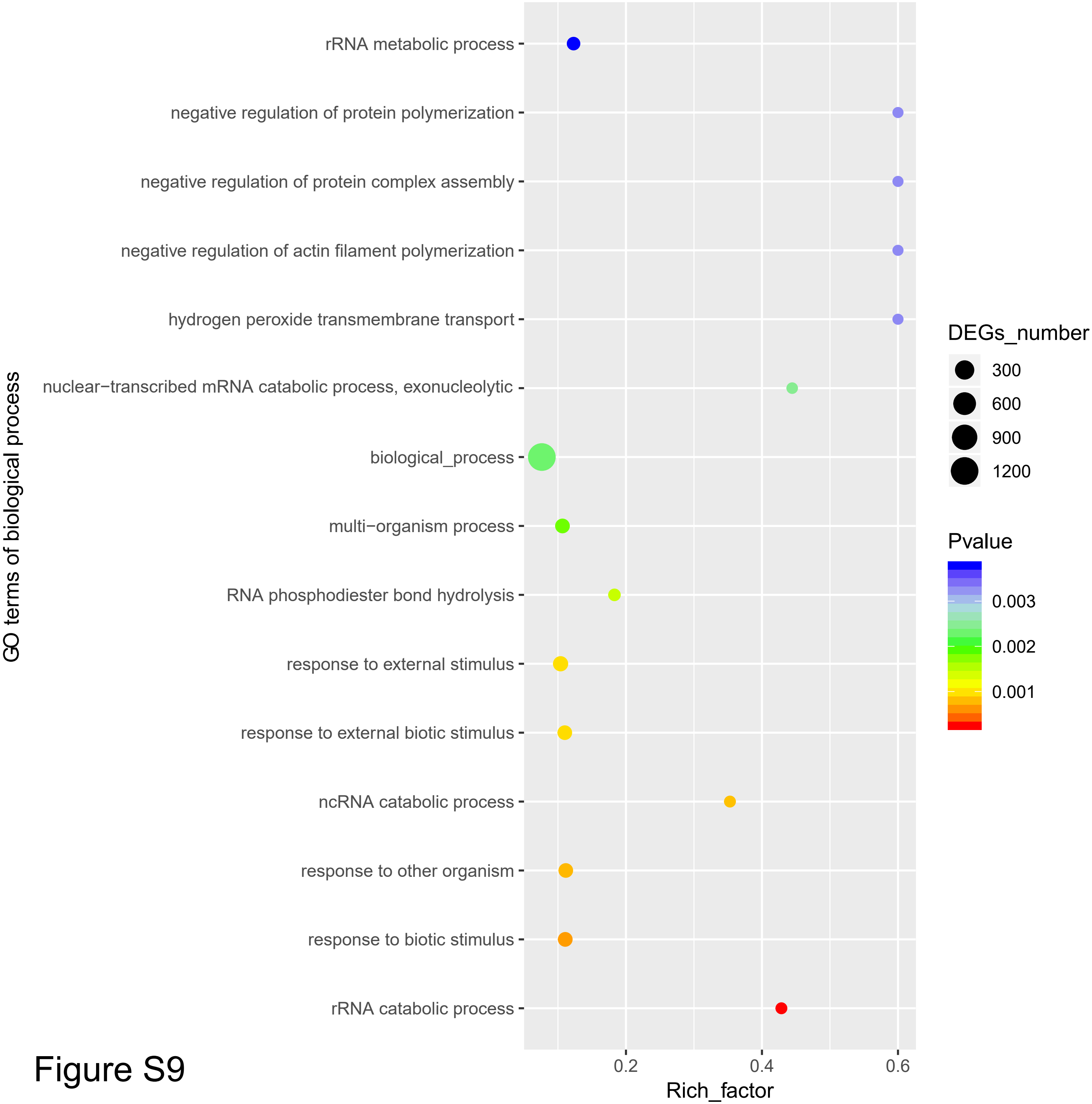
rRNA metabolic process
negative regulation of protein polymerization
negative regulation of protein complex assembly
negative regulation of actin filament polymerization
hydrogen peroxide transmembrane transport
nuclear−transcribed mRNA catabolic process, exonucleolytic
biological_process
multi−organism process
RNA phosphodiester bond hydrolysis
response to external stimulus
response to external biotic stimulus
ncRNA catabolic process
response to other organism
response to biotic stimulus
rRNA catabolic process
GO terms of biological process
0.2
0.4
0.6
Rich_factor
DEGs_number
300
600
900
1200
Pvalue
0.003
0.002
0.001

Figure S9

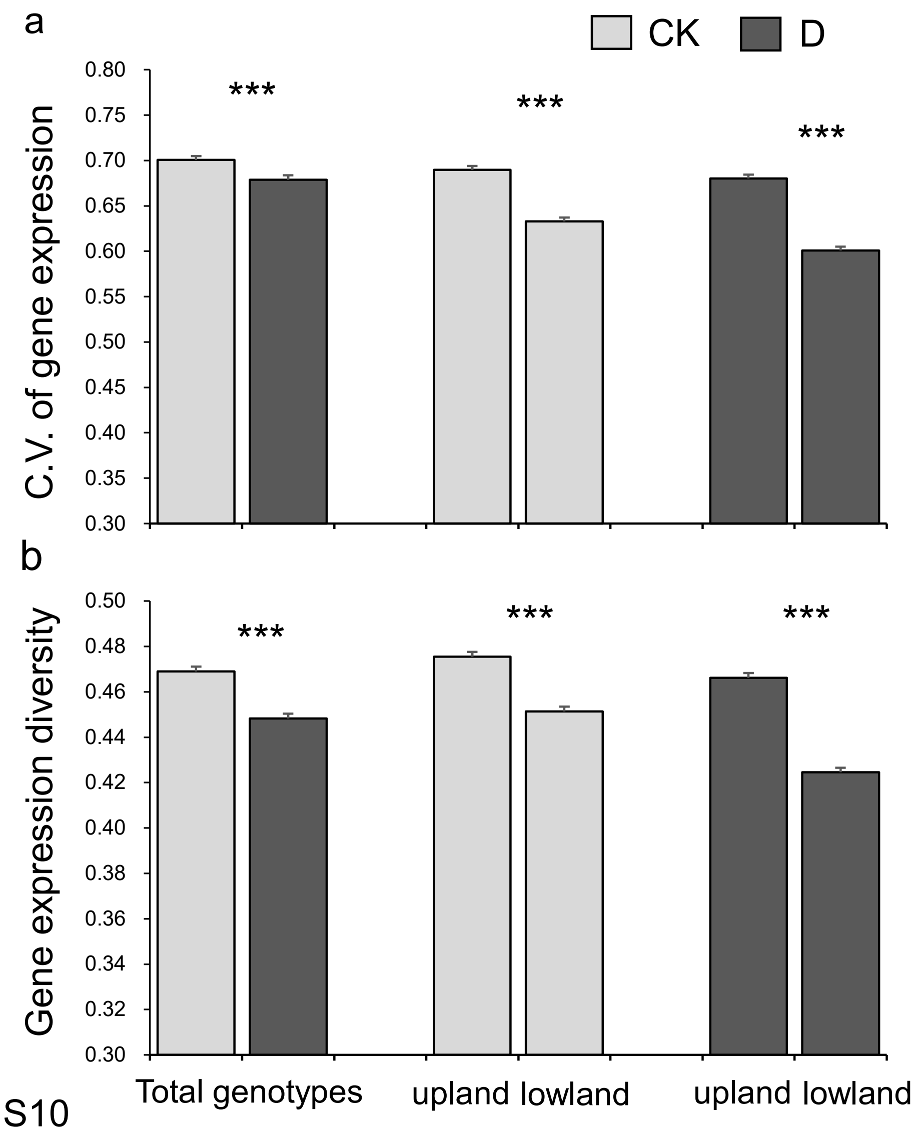
a
CK
D
C.V. of gene expression
0.80
0.75
0.70
0.65
0.60
0.55
0.50
0.45
0.40
0.35
0.30
***
***
***
b
Gene expression diversity
0.50
0.48
0.46
0.44
0.42
0.40
0.38
0.36
0.34
0.32
0.30
***
***
***
Total genotypes
upland lowland
upland lowland

Figure S10

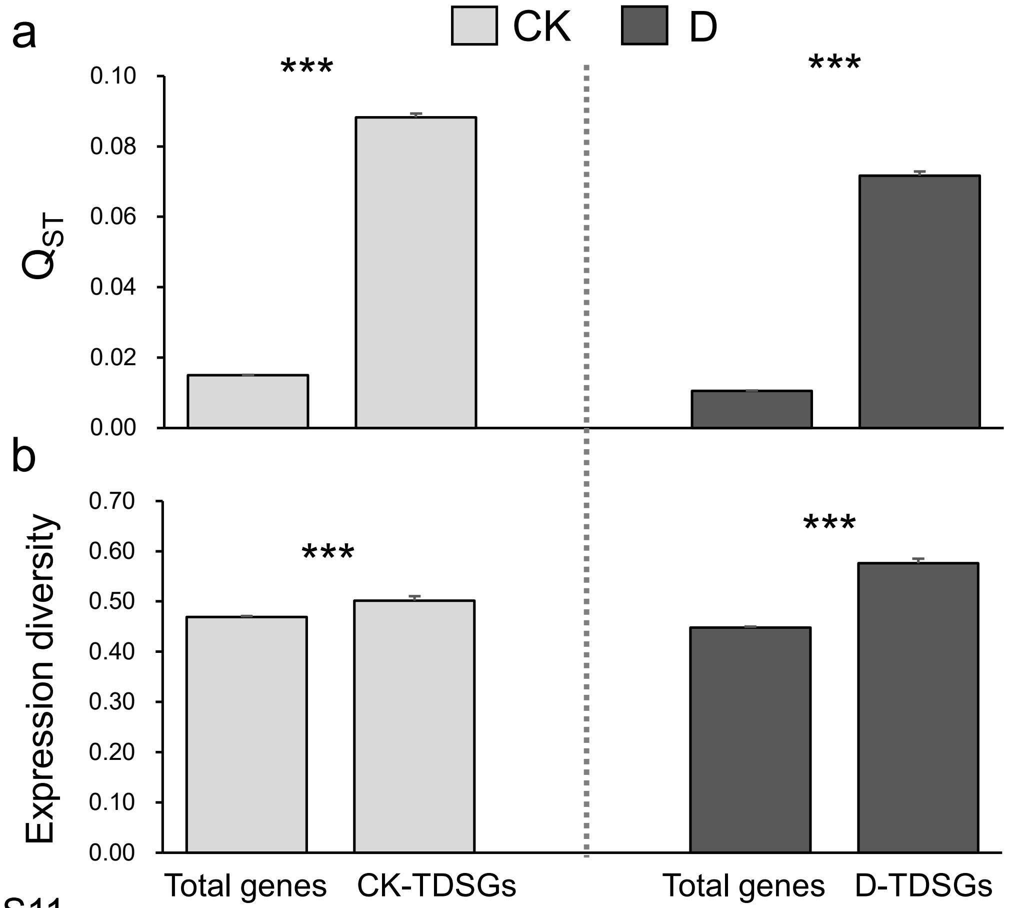

a
CK
D
***
***
0.10
0.08
0.06
0.04
0.02
0.00
Q_{ST}
b
0.70
0.60
0.50
0.40
0.30
0.20
0.10
0.00
Expression diversity
***
***
Total genes
CK-TDSGs
Total genes
D-TDSGs

Figure S11

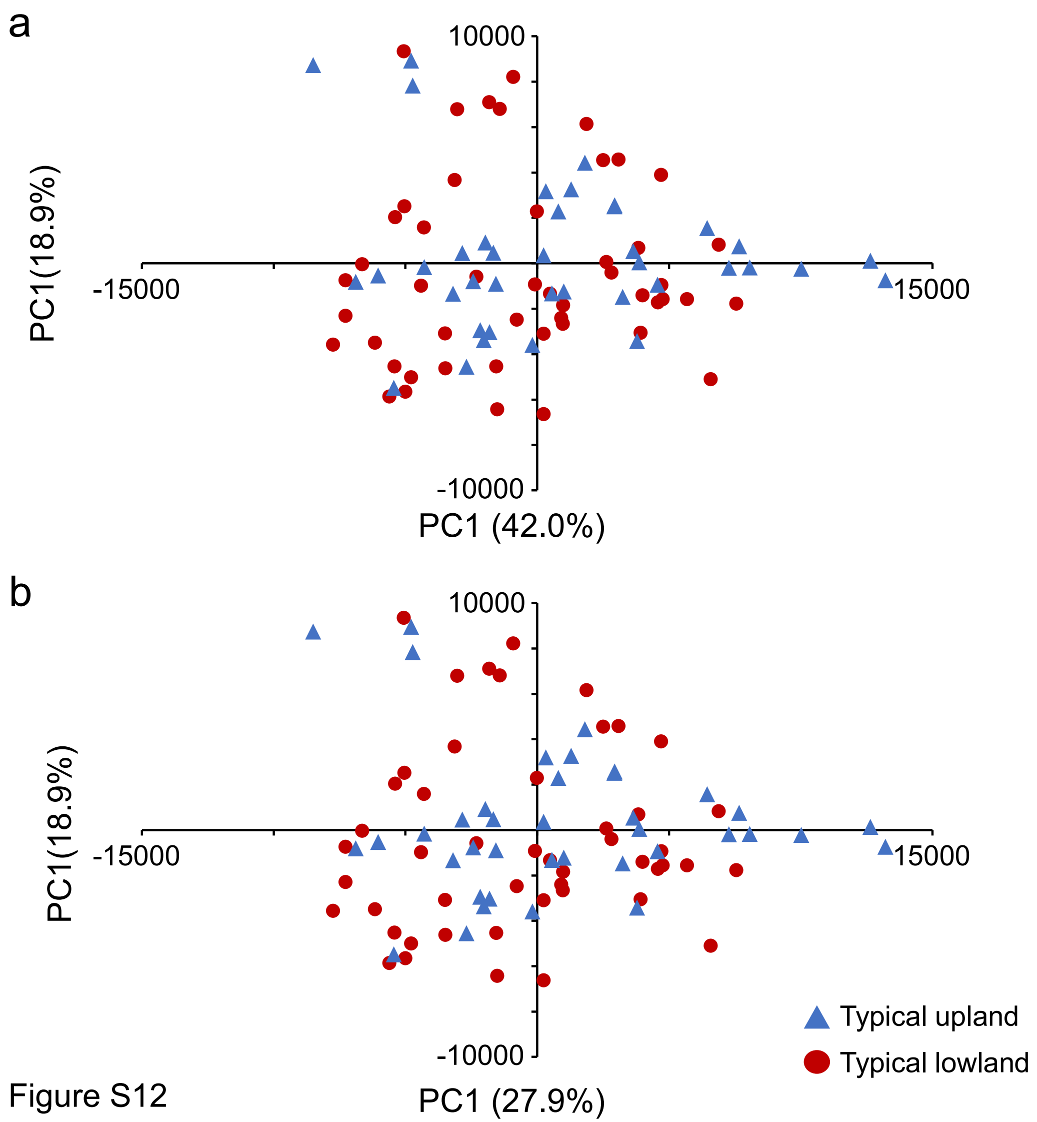
a
10000
-10000
-15000
15000
PC1(18.9%)
PC1 (42.0%)
b
10000
-10000
-15000
15000
PC1(18.9%)
PC1 (27.9%)
Typical upland
Typical lowland

Figure S12

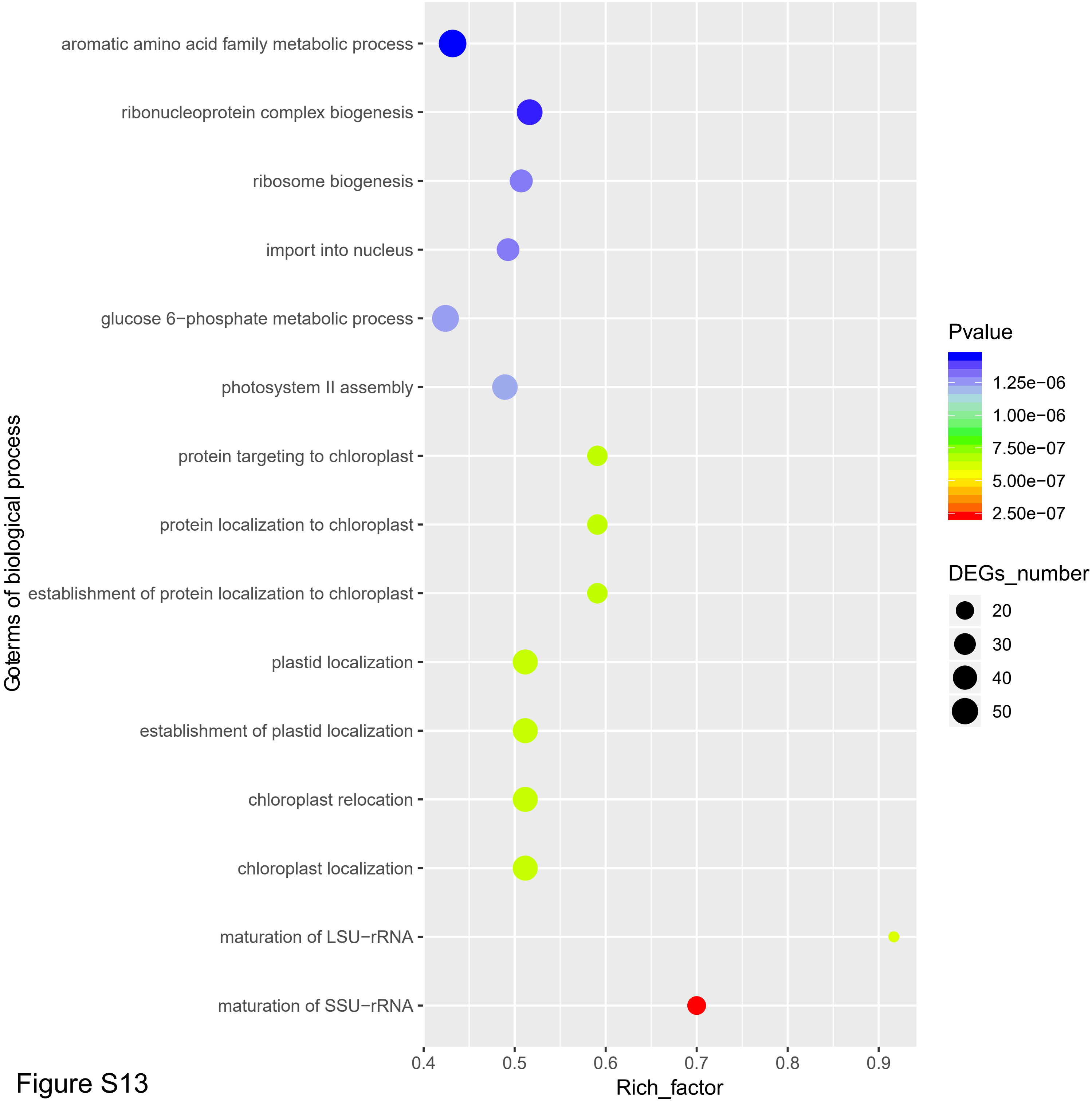

aromatic amino acid family metabolic process
ribonucleoprotein complex biogenesis
ribosome biogenesis
import into nucleus
glucose 6−phosphate metabolic process
photosystem II assembly
protein targeting to chloroplast
protein localization to chloroplast
establishment of protein localization to chloroplast
plastid localization
establishment of plastid localization
chloroplast relocation
chloroplast localization
maturation of LSU−rRNA
maturation of SSU−rRNA
Goterms of biological process
0.4
0.5
0.6
0.7
0.8
0.9
Rich_factor
Pvalue
1.25e−06
1.00e−06
7.50e−07
5.00e−07
2.50e−07
DEGs_number
20
30
40
50

Figure S13

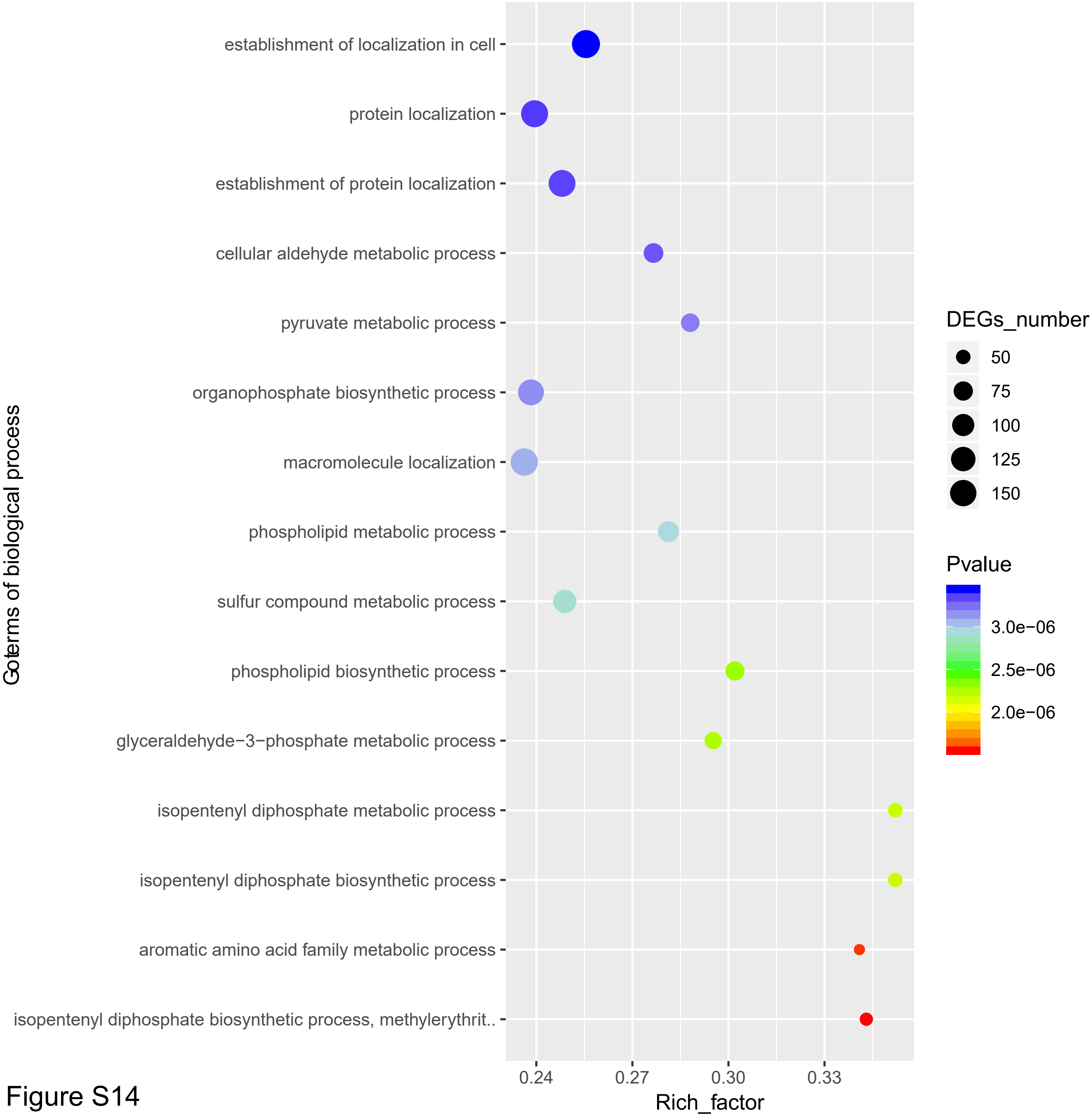

establishment of localization in cell
protein localization
establishment of protein localization
cellular aldehyde metabolic process
pyruvate metabolic process
organophosphate biosynthetic process
macromolecule localization
phospholipid metabolic process
sulfur compound metabolic process
phospholipid biosynthetic process
glyceraldehyde−3−phosphate metabolic process
isopentenyl diphosphate metabolic process
isopentenyl diphosphate biosynthetic process
aromatic amino acid family metabolic process
isopentenyl diphosphate biosynthetic process, methylerythrit..
Goterms of biological process
0.24
0.27
0.30
0.33
Rich_factor
DEGs_number
50
75
100
125
150
Pvalue
3.0e−06
2.5e−06
2.0e−06

Figure S14

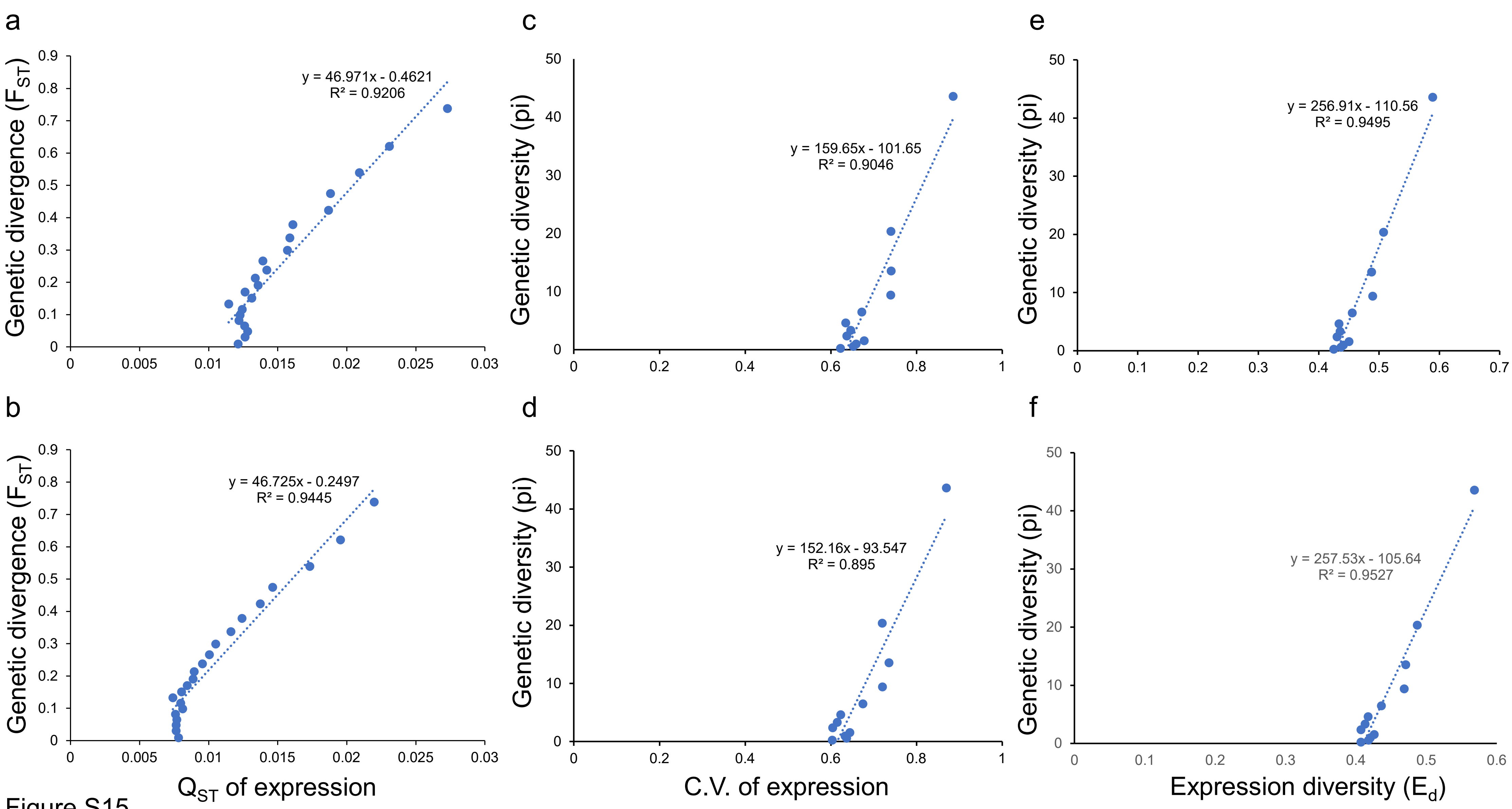

a
b
c
d
e
f
Genetic divergence (F_ST)
Genetic diversity (pi)
y = 46.971x - 0.4621
R² = 0.9206
y = 46.725x - 0.2497
R² = 0.9445
y = 159.65x - 101.65
R² = 0.9046
y = 152.16x - 93.547
R² = 0.895
y = 256.91x - 110.56
R² = 0.9495
y = 257.53x - 105.64
R² = 0.9527
Q_ST of expression
C.V. of expression
Expression diversity (E_d)

Figure S15

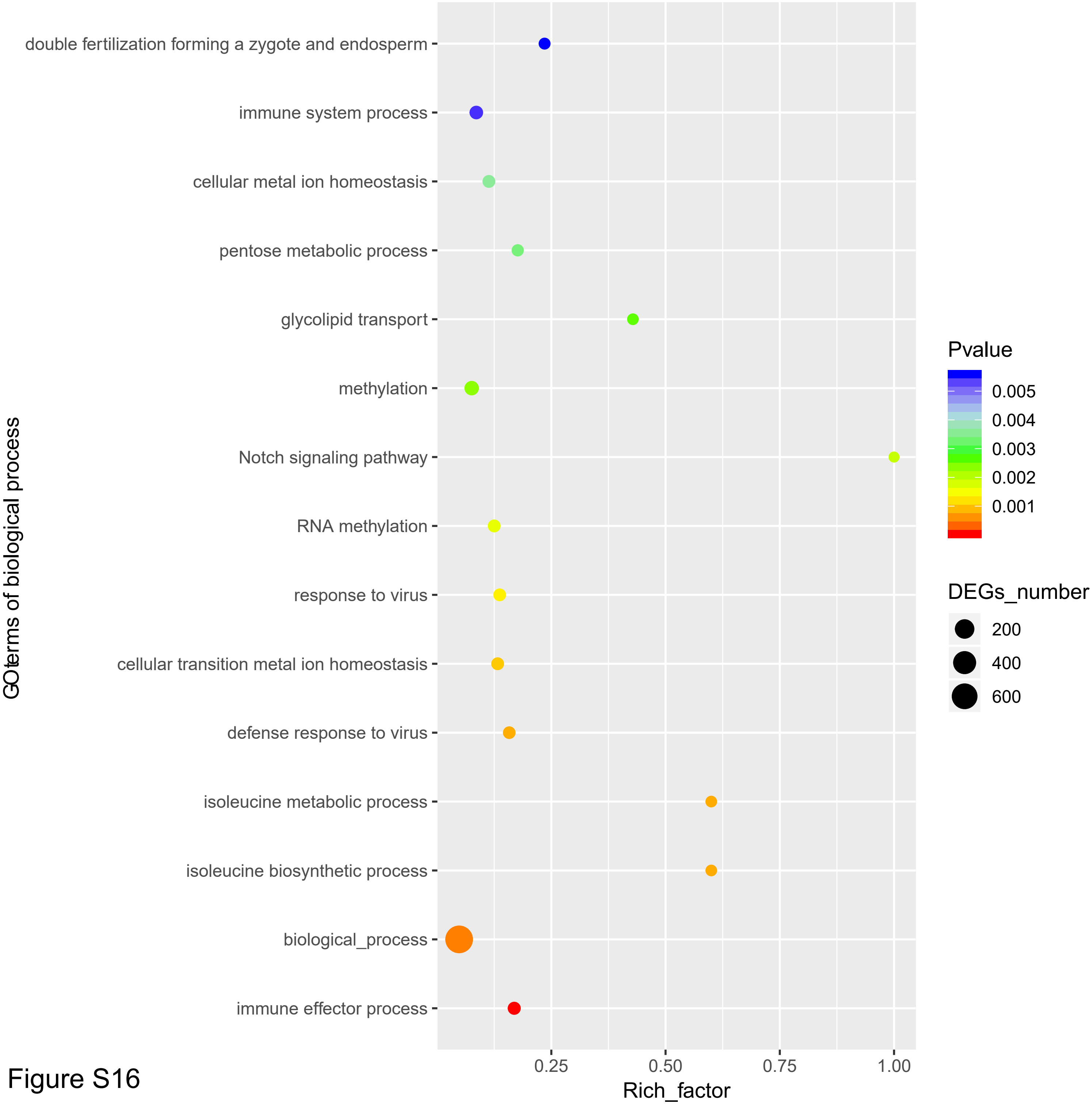

double fertilization forming a zygote and endosperm
immune system process
cellular metal ion homeostasis
pentose metabolic process
glycolipid transport
methylation
Notch signaling pathway
RNA methylation
response to virus
cellular transition metal ion homeostasis
defense response to virus
isoleucine metabolic process
isoleucine biosynthetic process
biological_process
immune effector process
GO terms of biological process
0.25
0.50
0.75
1.00
Rich_factor
Pvalue
0.005
0.004
0.003
0.002
0.001
DEGs_number
200
400
600

Figure S16

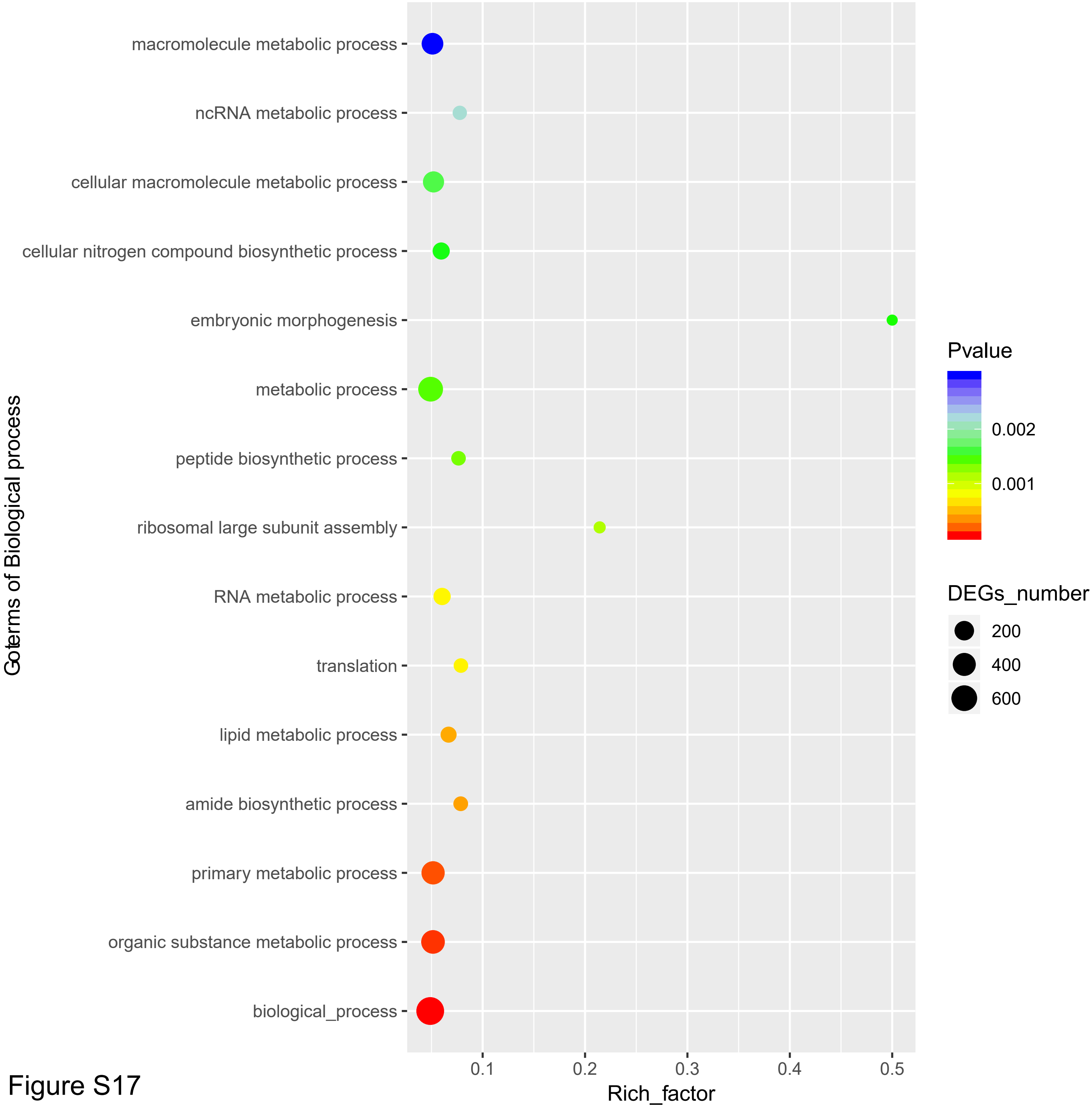

Goterms of Biological process
macromolecule metabolic process
ncRNA metabolic process
cellular macromolecule metabolic process
cellular nitrogen compound biosynthetic process
embryonic morphogenesis
metabolic process
peptide biosynthetic process
ribosomal large subunit assembly
RNA metabolic process
translation
lipid metabolic process
amide biosynthetic process
primary metabolic process
organic substance metabolic process
biological_process
0.1
0.2
0.3
0.4
0.5
Rich_factor
Pvalue
0.002
0.001
DEGs_number
200
400
600

Figure S17
